# Safeguarding biomedical AI: a critical scoping review of privacy-enhancing technologies, hybrid approaches, and deployment models

**DOI:** 10.3389/fdgth.2026.1726771

**Published:** 2026-08-17

**Authors:** Seha Ay, Umit Topaloglu, Wei Zhang

**Affiliations:** 1 Department of Biomedical Engineering, Wake Forest School of Medicine, Winston-Salem, NC, United States; 2 National Cancer Institute, Shady Grove, Rockville, MD, United States; 3 Department of Cancer Biology, Wake Forest School of Medicine, Winston-Salem, NC, United States

**Keywords:** biomedical AI, clinical deployment, differential privacy, federated learning, homomorphic encryption, hybrid PETs, privacy-enhancing technologies, secure multiparty computation

## Abstract

**Background:**

Biomedical artificial intelligence (AI) requires the integration of privacy-enhancing technologies (PETs) to safeguard sensitive clinical, imaging, and genomic data while preserving analytical utility.

**Objectives:**

This review critically and systematically maps applications of PETs across the biomedical AI lifecycle in accordance with PRISMA-ScR guidelines and evaluates their technical trade-offs, deployment feasibility, and residual risks.

**Methods:**

We systematically searched PubMed, IEEE Xplore, ACM Digital Library, and Scopus for studies published between 2015 and 2025. Eligible studies addressed differential privacy, federated learning, secure multiparty computation, homomorphic encryption, or hybrid approaches in biomedical AI. Data were charted on PET type, modality, lifecycle stage, utility metrics, privacy parameters, and deployment considerations. A critical appraisal rubric assessed threat-model adequacy, methodological clarity, reproducibility, privacy–utility transparency, and deployment realism. Additionally, we hand-searched major venues (USENIX Security, NeurIPS, AAAI) and screened Google Scholar for grey literature, applying de-duplication across sources.

**Results:**

We identified 87 studies spanning clinical decision support, genomics, and medical imaging. From 25,761 initial records, 3,754 underwent title/abstract screening and 1,968 underwent full-text assessment. PETs demonstrated distinct strengths and limitations: differential privacy provided provable guarantees but reduced performance on imbalanced data; federated learning improved data access but remained vulnerable to gradient leakage; and cryptographic methods ensured confidentiality at high computational cost. Synthetic data generation supported privacy-conscious data sharing and benchmarking but remained sensitive to disclosure risk, fidelity loss, and subgroup representation. Hybrid and emerging approaches, including trusted execution environments, zero-knowledge proofs, and privacy-preserving transformer architectures, mitigated composability gaps yet lacked full end-to-end assurance. Case studies at hospital and biobank scale illustrated practical feasibility and infrastructure demands.

**Conclusions:**

Situating PETs within technical and operational contexts clarifies their capabilities, limitations, and deployment challenges. Residual risks persist, including fairness concerns, inference-time leakage, and overreliance on PETs as compliance proxies. Sustained technical innovation and institutional governance remain essential for the trustworthy integration of PETs in biomedical AI.

## 1 Introduction

Biomedical AI creates a practical tension: the data needed to train useful models are often the same data that are most difficult to share safely. Clinical records, imaging studies, genomic profiles, and wearable data can improve prediction and decision support, but they may also reveal patient identity, disease status, family risk, or institutional characteristics. Privacy-enhancing technologies (PETs) are designed to reduce these risks while allowing data analysis or model development to continue. In simple terms, PETs aim to answer a central question for biomedical AI: how can institutions learn from sensitive data without unnecessarily exposing the people represented in those data?

The growing integration of artificial intelligence (AI) into biomedical research and clinical practice has ushered in transformative opportunities in early detection, diagnosis, prognosis, treatment personalization, follow-up, and public health surveillance. Machine learning models trained on vast biomedical datasets such as electronic health records (EHRs), genomic and epigenetics profiling, medical imaging, and digital biomarkers can identify intricate patterns often overlooked by human experts (1). However, these capabilities also introduce significant privacy risks. Biomedical data often contain thousands of correlated, high-dimensional features, which can enable re-identification even after de-identification. Genetic sequences, imaging biomarkers, and rare disease phenotypes may serve as near-unique identifiers of an individual. Moreover, such data are clinically sensitive, reflecting health conditions and outcomes that require strict protection of privacy, confidentiality, and responsible stewardship (2).

Historically, healthcare data protection has relied on access control, encryption, and adherence to legal frameworks such as the Health Insurance Portability and Accountability Act (HIPAA) and the General Data Protection Regulation (GDPR). While these policies provide important structural safeguards, they are insufficient against adversarial threats that emerge once sensitive data are used to train machine learning models (3, 4). Deep learning models can memorize and inadvertently leak private information, creating new attack surfaces beyond traditional data breaches (5). Studies show that even anonymized datasets or black-box model outputs can be exploited through membership inference, model inversion, and attribute inference attacks (6, 7).

In response, the field of privacy-preserving machine learning has developed algorithmic defenses designed to mitigate such risks while maintaining model utility. These include Differential Privacy (DP) (8), Federated Learning (FL) (9), Secure Multi-Party Computation (SMPC), Homomorphic Encryption (HE) and synthetic data generation (10). Yet these privacy-enhancing technologies (PETs) differ in their coverage of threats, ease of deployment, and performance trade-offs. Their guarantees are frequently misunderstood or misapplied, particularly in biomedical contexts where operational constraints, interpretability, and clinical reliability matter. For instance, some PETs protect data during training but fail to safeguard models once deployed in practice (4, 11).

To contextualize the evolution of biomedical data privacy, we reference foundational works published before 2015 (e.g., 8, 14, 19, 21). These are included only to frame the emergence of privacy-enhancing technologies, whereas the formal evidence synthesis in this critical scoping review is limited to studies published between 2015 and 2025.

Unlike prior narrative reviews, this work applies a critical scoping review (CSR) methodology to systematically map PET applications in biomedical AI. A scoping design is appropriate because the field is broad, heterogeneous, and evolving rapidly across machine learning, cryptography, and biomedical domains. The “critical” element reflects our appraisal of not only where PETs are applied but also the adequacy of their threat models, reproducibility, and deployment realism.

This review offers a technical synthesis of PETs within biomedical AI, mapping specific methods to stages of the model lifecycle, with emphasis on both training and post-deployment phases. We assess each technology's mechanisms, limitations, and application boundaries, while highlighting how privacy risks evolve over time and under different adversarial assumptions. We also consider system-level infrastructure and deployment challenges, recognizing that technical solutions must be evaluated in relation to clinical workflows and governance structures (12, 13).

The objectives of this critical scoping review are framed as four research questions (RQs):

RQ1: How do privacy threats evolve across the biomedical AI model lifecycle, and what adversarial models are most relevant?
RQ2: What privacy-enhancing technologies have been applied in biomedical AI, how do their guarantees and limitations map to different lifecycle stages, and what patterns emerge across data modalities and clinical domains?
RQ3: What infrastructure and operational factors influence the feasibility of PET deployment in biomedical research and clinical environments?
RQ4: What residual risks and critical challenges remain unresolved despite current PET adoption, and how robust and transparent is the underlying evidence base for guiding future research and governance?

By structuring the review around these questions, we clarify both the breadth of PET adoption and the strength of supporting evidence, setting the stage for critical appraisal of technical claims and operational feasibility.

## 2 Background and threat models

Before comparing individual PETs, it is useful to clarify where privacy risks arise. Biomedical AI systems do not expose information only when raw data are shared. Leakage can occur during data preparation, model training, model release, inference, explanation, or downstream integration into clinical software. A lifecycle view therefore helps distinguish what type of adversary is relevant at each stage, what information may be exposed, and which PETs are appropriate for that specific risk.

### 2.1 Biomedical data characteristics

**Figure 1:**
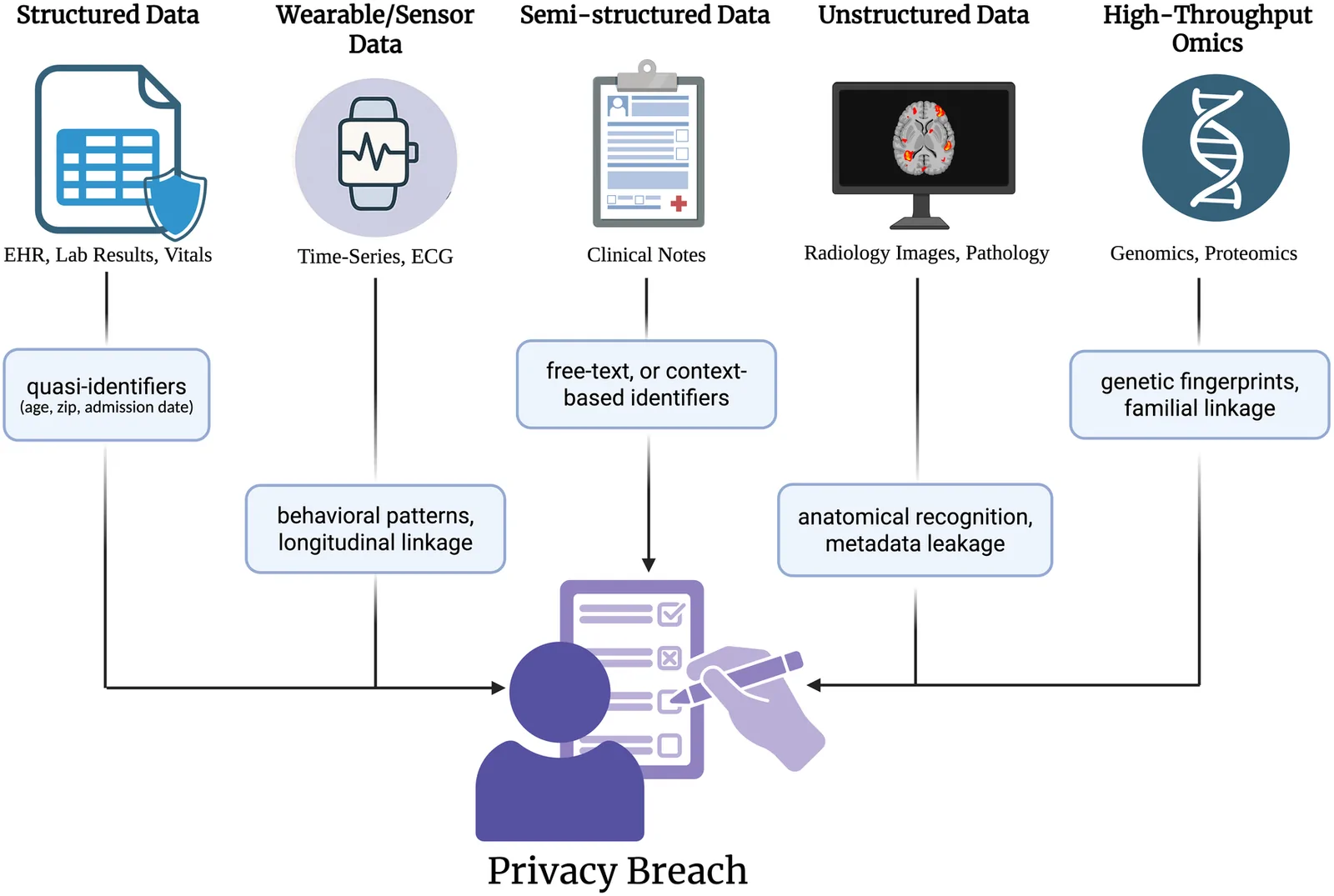
Different data types such as structured records, wearables, clinical notes, imaging, and genomics carry unique identifiers and latent features that can lead to re-identification, even after de-identification efforts. Created in BioRender. Ay, S. (2026) https://BioRender.com/rksoim3, licensed under CC BY.

Biomedical data is distinguished from most other AI domains by its high complexity, sensitivity, and dimensionality. It spans multiple modalities, including structured formats such as electronic health records (EHRs), semi-structured clinical narratives, unstructured sources like medical imaging, and increasingly, high-throughput omics and longitudinal patient data. Each modality presents distinct privacy challenges shaped by both clinical and technical contexts of collection, storage, and analysis (Figure 1).

Structured clinical data, such as diagnosis codes, medication records, laboratory results, and vital signs, is sometimes perceived as less sensitive due to its tabular format. However, even small subsets, when linked with quasi-identifiers such as age, ZIP code, or admission date, can enable high-confidence re-identification (2). Genomic data poses an even greater challenge: even when explicit identifiers are removed, DNA sequences remain inherently unique, can reveal kinship, and expose hereditary predispositions, rendering conventional de-identification ineffective (14, 15).

Unstructured and semi-structured modalities, including radiology images, histopathology slides, and clinical notes, add further complexity. Latent features in these formats are difficult to anonymize without impairing clinical utility. For example, craniofacial anatomy in head CT scans or handwritten annotations in pathology images can encode identity (11). Free-text clinical notes often contain direct identifiers or contextually revealing phrases that disclose sensitive patient details. Large-scale radiology datasets have also been shown vulnerable to adversarial re-identification, where identifiable features were reconstructed from metadata and anatomical patterns even after pixel-level identifiers were removed.

Risks increase when biomedical datasets are longitudinal, multimodal, and heterogeneous. While these datasets support advanced predictive modeling, they also heighten the potential for linkage and attribute inference attacks, especially when multiple data sources are combined. The proliferation of wearable devices and continuous monitoring systems contributes high-frequency, high-resolution time-series data, embedding unique behavioral or physiological signatures that are resistant to anonymization without substantial loss of diagnostic value (16).

Sparsity presents an additional and often underestimated risk. Data representing underrepresented populations, rare diseases, or low-resource settings is typically limited in volume yet highly identifiable. Without privacy mechanisms adapted to skewed distributions, these groups may receive inadequate protection. Synthetic or transformed datasets that preserve density patterns may further heighten identifiability for rare cohorts (10). Confirmed cases remain limited, but linkage attacks on de-identified registries and the uniqueness of rare-disease records suggest that synthetic cancer registries could be vulnerable. Recent evaluations of synthetic data further report measurable risks of membership inference and the ability to identify unique individuals, underscoring that synthetic releases do not eliminate linkage threats for uncommon records (17, 18).

### 2.2 Evolution of privacy safeguards in biomedical AI

Concerns about privacy in biomedical data predate artificial intelligence and are rooted in longstanding principles of confidentiality, ethical oversight, and trust between patients and healthcare providers. Historically, safeguards were primarily institutional and legal. Data access was restricted through IRB protocols, protected by perimeter-based security architectures, and regulated under frameworks such as HIPAA and the Common Rule (12). These measures emphasized structural protections, relying on access control, contractual agreements, and de-identification practices to preserve confidentiality.

However, technical research revealed the fragility of these protections. A widely cited example involved the re-identification of Massachusetts Governor William Weld, whose anonymized health records were linked to voter registration data using quasi-identifiers, demonstrating that removing direct identifiers was insufficient (19). Subsequent studies confirmed that combinations of variables such as age, sex, and ZIP code could uniquely identify many individuals (14), indicating that the risk extends beyond rare scenarios.

The challenge intensified with the rise of large-scale biomedical repositories such as the UK Biobank, The Cancer Genome Atlas (TCGA), and the All of Us initiative. These platforms aggregate high-dimensional, longitudinal, and cross-institutional data, substantially increasing the risk of re-identification and secondary misuse (15). Although no confirmed large-scale re-identification has occurred, genomic datasets have been linked back to individuals under specific conditions. For example, Gymrek et al. inferred surnames from personal genomes using Y-chromosome STRs and genealogical databases, then triangulated identity using metadata such as age and state (20).

With the advent of machine learning, privacy risks expanded: adversaries can exploit trained models through membership inference, model inversion, or latent feature leakage (5, 7). In response, privacy-preserving strategies evolved along three trajectories. First, formal privacy mechanisms such as differential privacy (DP) and homomorphic encryption (HE) introduced mathematical bounds on individual influence within aggregated outputs (8). Second, approaches emphasizing data minimization and decentralization, including federated learning (FL) and secure multiparty computation (SMPC), reduced the need for raw data sharing in collaborative modeling (4, 9). Third, scholars advanced contextual privacy frameworks, recognizing that privacy depends not only on algorithms but also on social and clinical contexts (3, 21). In biomedical AI, this requires aligning technical safeguards with domain-specific norms such as informed consent, appropriate reuse of clinical records, and the preservation of patient trust.

Despite these innovations, practical limitations persist. DP can significantly reduce utility in datasets with small or underrepresented subgroups, as noise injection disproportionately affects rare event detection. FL, while reducing data centralization, requires substantial coordination infrastructure and assumes a level of model uniformity that is often unrealistic in heterogeneous healthcare environments (11, 13). These limitations illustrate the enduring privacy–utility trade-offs central to biomedical AI, examined further in Sec. 6.2.

Privacy in biomedical AI remains a multi-layered, evolving challenge encompassing technical, ethical, institutional, and sociopolitical dimensions. Safeguards must be adaptive, aligning with changes in data characteristics, deployment settings, stakeholder relationships, and regulatory frameworks. This broader perspective underscores the importance of identifying where privacy threats arise and how PETs can be aligned to those evolving risks.

To make this mapping explicit, Table 1 summarizes representative threat models across the biomedical AI lifecycle. Here, attacker capability refers to the level of access available to an adversary, ranging from external black-box querying to insider or white-box access to model internals, gradients, logs, or deployment infrastructure. The listed defenses should be interpreted as context-dependent countermeasures rather than universal solutions. A defense marked as mature may be well established for a particular threat model, but its effectiveness still depends on implementation quality, adversary strength, deployment setting, and whether it is combined with complementary safeguards.

**Table 1:** Threat model matrix in biomedical AI.

Stage	Attacker capability	Goal	Exposed signals	Suitable defenses
Training	Insider, white-box access	Membership inference (7), model inversion (6), property inference (22)	Gradients, activations, logs	DP-SGD (23) ✓; Secure aggregation (24) ✓; SMPC (25) ✓; TEEs (26) ✓; Access control & audit (4) ✓
Training	Malicious or Byzantine participant	Data poisoning (27), backdoor insertion (27)	Gradient updates, model merges	Robust aggregation (28) ✓; Anomaly detection (28) ✓; Byzantine-resilient FL (28) ∼; DP (23) ∼
Training	Supply-chain adversary	Malicious libraries (29), pre-trained weights (29), or CI/CD artifacts (29)	Containers, model checkpoints, build pipeline	TEEs (26) ✓; Policy-as-code (29) ✓
Inference (online)	External black-box querier	Extraction (5), inversion (6), membership inference (7)	Logits, probabilities, explanations, timing	Post-hoc DP (30) ✓; Logit clipping (30) ✓; Query budgets & rate-limits (30) ✓; HE for inference (31) ∼
Inference (partner access)	Gray-box collaborator	Property inference (22), model theft (11)	Batched queries, partial internals	HE/TEEs (26, 32) ✓; Access scopes (4) ✓; Knowledge distillation (11) ∼
Model release	Offline analyst	Training data reconstruction (5)	Weights, embeddings, documentation	DP-trained models (23) ✓; Weight publishing policy (4) ✓; Restricted embedding release (30) ✓
Imaging pipeline	Adversarial example crafter	Leakage of PHI (33)	Grad-CAM, SHAP, perturbed inputs	Regularization (33) ∼; Limited explanation fidelity (34) ∼; DP on explanations (34) ∼

✓ = mature defense; ∼ = partial/experimental defense.

This matrix synthesizes major threat models in biomedical AI across the lifecycle, mapping attacker capabilities to goals, exposed signals, and representative defenses. Defenses are representative rather than exhaustive. A check mark indicates a relatively established mitigation under the stated assumptions, whereas a tilde indicates a partial, experimental, or context-dependent mitigation. Neither symbol implies complete protection across all biomedical deployments.

### 2.3 Privacy threats across the biomedical AI lifecycle

#### 2.3.1 Lifecycle framing and taxonomy

**Figure 2:**
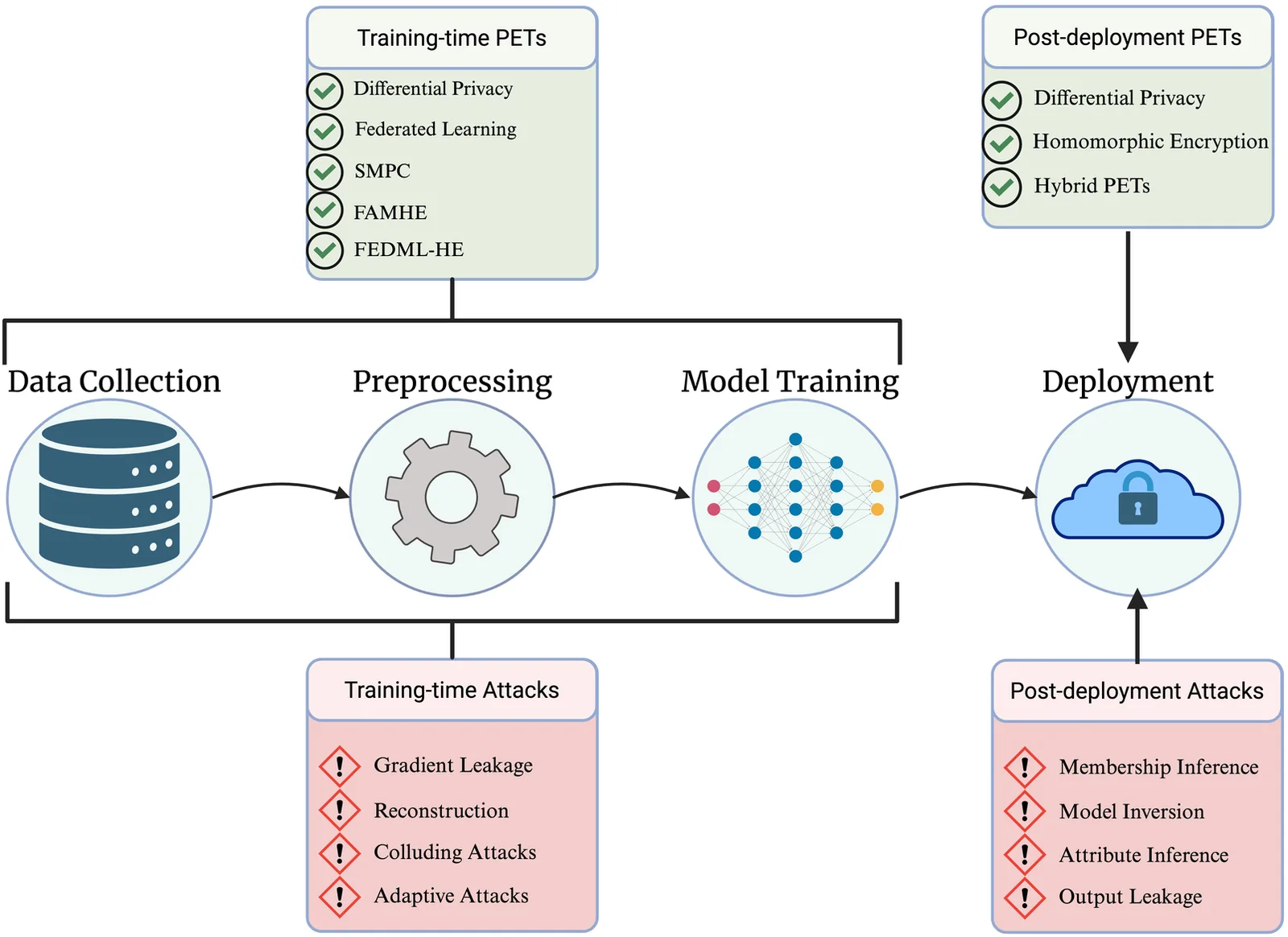
Life cycle of a biomedical AI model showing potential adversarial threats and privacy-enhancing technologies (PETs) suited to training and post-deployment stages. Created in BioRender. Ay, S. (2026) https://BioRender.com/w6upf9r, licensed under CC BY.

Building on Sections 2.1 and 2.2, privacy risks in biomedical AI can be better understood within the stages of the model lifecycle. Rather than viewing privacy as a single challenge, we distinguish between vulnerabilities during model training and those emerging after deployment (Table 1, Figure 2). This lifecycle-aware framing highlights how distinct adversarial strategies exploit different exposure points and clarifies why PETs must be matched to the phase they protect.

Table 1 synthesizes attacker capabilities, exposed signals, and defenses across training and deployment. It shows that some defenses, such as differential privacy or secure aggregation, are relatively established under specific assumptions, whereas others, including adversarial robustness and explanation-level protections, remain partial or experimental in biomedical settings. No single PET offers complete protection; defenses must be tailored to both adversary capability and lifecycle stage. Lifecycle-aware design therefore remains essential to avoid assuming that any one method ensures universal coverage.

Figure 2 complements this matrix by mapping adversarial threats and PETs across training and deployment phases. It illustrates how risks evolve as models move from development to clinical use, underscoring the need to align PET selection with the stage of vulnerability.

#### 2.3.2 Training-time threats in biomedical AI

**Training-time attacks** occur when models directly or indirectly access sensitive biomedical data. Adversaries may observe or manipulate internal parameters, gradients, or communication streams, especially in collaborative learning. Examples include reconstruction or gradient-leakage attacks, where patient-level features are recovered from shared updates (35, 36), and collusion, where malicious participants infer attributes from partial information. Such attacks exploit model over-parameterization, memorization tendencies, or insecure communication. Mitigations include secure aggregation, training-time differential privacy, and cryptographic methods such as SMPC and HE (4).

Biomedical training data are both acutely sensitive and structurally vulnerable, including genomic sequences, mental-health records, and longitudinal imaging data. Their small size yet high identifiability make them prone to leakage in decentralized learning, where updates or intermediate representations are exchanged across sites.

Gradient-based leakage is a major threat. In federated-learning settings, where institutions share gradients rather than raw data, unprotected updates can reveal sensitive information. Under small batches and unmasked gradients, researchers have reconstructed input images and structured records from updates (35, 36). In more realistic deployments, local SGD, batch averaging, or inter-client heterogeneity reduce effectiveness, but leakage risks persist (37, 38).

Collusion, bias leakage, and downstream privacy impacts represent broader classes of training-time risks. In semi-trusted collaborations, partners may deviate from SMPC or FL protocols, allowing malicious actors to combine partial information across rounds and infer sensitive attributes about patients whose data reside elsewhere (4). Model updates can also reveal structural biases, such as the underrepresentation of certain populations or rare disease subtypes, inadvertently exposing disparities across institutions (16). Beyond these indirect leaks, training-time exposure of genomic or phenotypic data can have severe consequences, compromising not only individual privacy but also revealing hereditary information about relatives who never consented to data sharing (11).

Beyond data leakage, training pipelines face supply-chain threats, where adversaries compromise workflows via malicious libraries, tampered pre-trained weights, or poisoned containers in CI/CD pipelines. These bypass gradient-level defenses and enable manipulation or exfiltration at the system level. Mitigations include SBOMs, reproducible builds, cryptographic code-signing, and execution inside TEEs to preserve provenance and integrity (29, 39).

#### 2.3.3 Post-deployment threats in biomedical AI

**Post-deployment attacks** occur once models are exposed for inference through APIs, decision-support tools, or embedded software. Adversaries cannot access training data or parameters but can issue queries and analyze outputs. Documented examples include membership inference [testing if specific patients contributed to training (7)], model inversion [reconstructing input features (6)], and property inference [learning aggregate trends (22)]. These attacks exploit statistical artifacts rather than direct data access. Mitigations such as post-training differential privacy, output perturbation, and confidence masking help but often reduce accuracy.

Membership-, inversion-, and property-inference attacks collectively exploit statistical artifacts of trained models rather than direct data access. Membership inference is particularly problematic in rare-disease models, where class imbalance increases memorization of unique profiles, enabling adversaries to probe predictions and infer patient participation, thereby breaching confidentiality (7). Model inversion reconstructs sensitive input features such as medical images or patient traits (6), while property inference targets aggregate characteristics, revealing dataset-level information such as disease prevalence or demographic composition and risking community-level privacy violations (22). These attacks can be amplified through repeated querying of exposed APIs or decision-support interfaces, where adaptive strategies accumulate partial information across interactions to bypass defenses like output rounding or logit clipping, particularly in systems returning detailed outputs (5).

Adversarial examples create further risk in biomedical imaging, where imperceptible perturbations mislead models and may expose protected health information. Interpretability tools such as SHAP and LIME can inadvertently reveal patient attributes by reconstructing salient regions. While defenses such as adversarial training, input smoothing, or applying differential privacy to explanations exist, these remain partial and experimental (33, 34).

Biomedical deployments also face indirect leakage through software behavior. AI tools integrated into EHR systems or mobile-health apps may disclose private data via interface feedback or background synchronization. Such channels show that privacy must be protected across the entire sociotechnical system, not only within algorithms.

Taken together, training-time and post-deployment threats show that privacy risks in biomedical AI are dynamic and context dependent. The same dataset can be vulnerable in distinct ways depending on exposure during training or inference. Biomedical factors such as rare-disease prevalence, genomic uniqueness, and heterogeneous workflows amplify both individual- and community-level risks. No single defense covers all attacks, making lifecycle-aware strategies essential. This recognition grounds the later critical evaluation of PETs (Sec. 4).

## 3 Methods

### 3.1 Protocol and framework

This review was conducted in accordance with the PRISMA Extension for Scoping Reviews (PRISMA-ScR), which provides a structured methodology for synthesizing heterogeneous evidence. A scoping design was chosen over a systematic review because the literature on privacy-enhancing technologies (PETs) in biomedical artificial intelligence (AI) is diverse in scope, spans multiple disciplines, and includes emerging methods that have not yet been clinically validated. The approach is “critical” in that it does not merely catalogue applications of PETs but also appraises the adequacy of reported threat models, the transparency of privacy-utility trade-offs, and the feasibility of deployment in biomedical contexts.

### 3.2 Information sources and search strategy

A comprehensive search was undertaken across four major bibliographic databases: PubMed, IEEE Xplore, ACM Digital Library, and Scopus. The timeframe of interest was January 1, 2015 through August 31, 2025, reflecting the period in which most modern PET applications in biomedical AI have been reported; final reference-library reconciliation was completed in September 2025. To provide historical grounding, works published between 2000 and 2014 were also considered when they represented foundational advances in biomedical privacy, such as re-identification studies, ethical frameworks, or the introduction of formal privacy models.

Search strings combined three concept blocks: (i) PET methodologies, including differential privacy, federated learning, secure multiparty computation, homomorphic encryption, synthetic data generation, trusted execution environments, and zero-knowledge proofs; (ii) biomedical domains, including electronic health records, medical imaging, genomics, biobanks, wearable sensors, and clinical decision support; and (iii) privacy or deployment outcomes, including re-identification, membership inference, model inversion, attribute inference, privacy leakage, deployment, and clinical implementation. Searches were adapted to the syntax of each database, and the database-specific search strings are provided in Appendix A.

To complement database searching, the proceedings of leading conferences, including USENIX Security, NeurIPS, and AAAI, were hand-searched for applied PET studies in biomedical contexts. In addition, grey literature was identified through a targeted Google Scholar sweep to capture system implementations, deployment reports, and relevant preprints (including arXiv) that were not indexed in standard databases. Finally, the reference lists of included studies were screened to ensure comprehensive coverage of the field.

In total, the searches identified 25,761 records across databases. After applying filters, removing duplicates, and excluding out-of-domain works, 3,754 studies were retained for title/abstract screening. Of these, 1,968 full-text articles were assessed for eligibility, leading to the inclusion of 87 studies in the final synthesis.

### 3.3 Eligibility criteria

Studies were included if they addressed the design, evaluation, or deployment of PETs in biomedical AI. Eligible domains encompassed structured and unstructured clinical data, genomic and multi-omics resources, medical imaging, longitudinal health records, and high-frequency data from wearable devices. Eligible PETs included differential privacy, federated learning, secure multiparty computation, homomorphic encryption, hybrid frameworks, synthetic data generation, trusted execution environments, and zero-knowledge proofs.

Exclusion criteria were applied to omit purely theoretical cryptographic contributions without biomedical applicability, non-peer-reviewed works lacking methodological detail, and publications outside the 2000–2025 timeframe. During screening, further exclusions included inaccessible full texts, works outside the timeframe despite initial indexing, and duplicate preprints or commentaries without additional methodological contribution. In keeping with the scoping review design, both peer-reviewed articles and preprints were considered when they provided substantive insights into biomedical use cases.

### 3.4 Study selection

**Figure 3:**
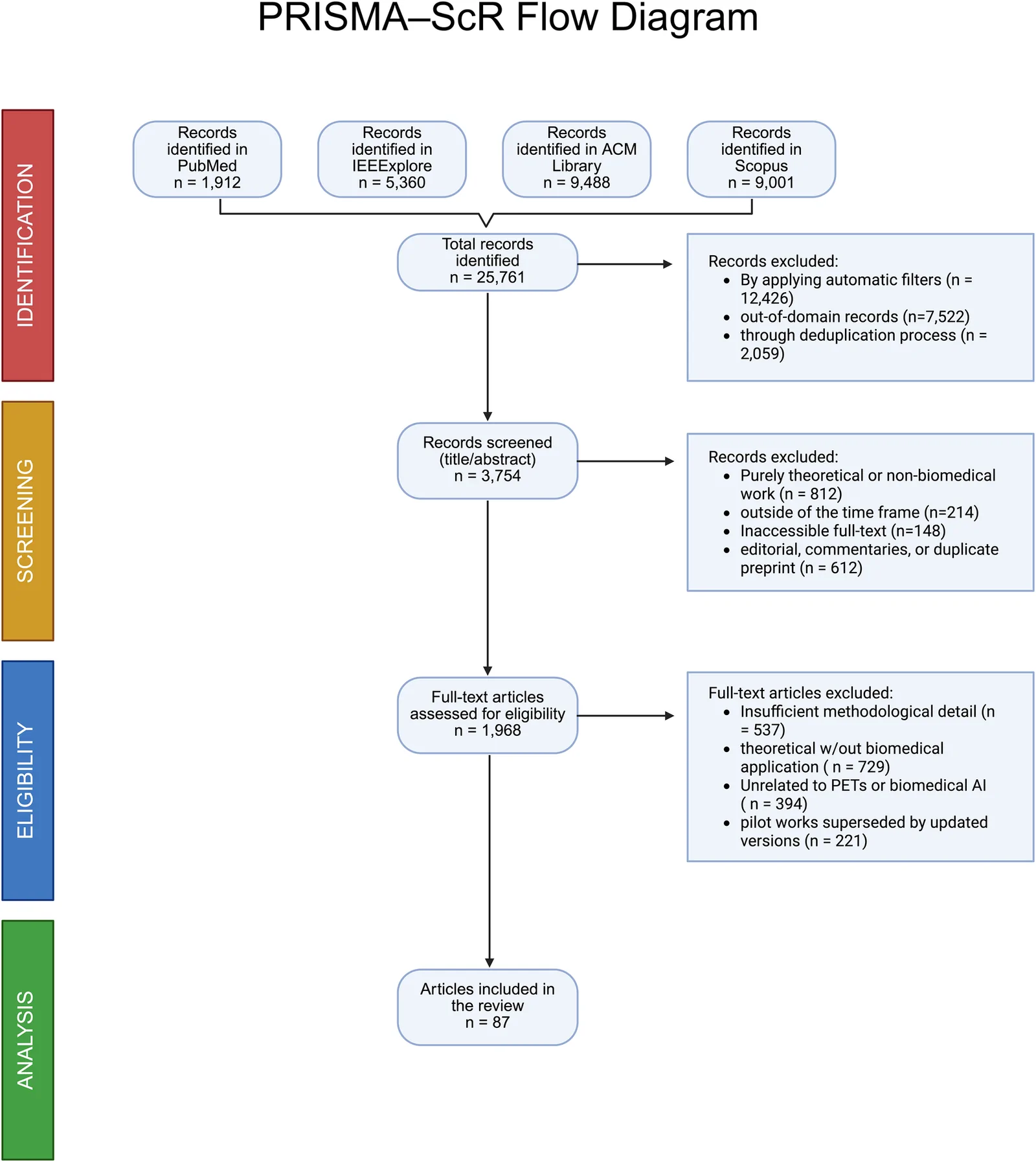
PRISMA-ScR flow diagram of the study identification, screening, eligibility, and inclusion process after final reference-library reconciliation. Created in BioRender. Ay, S. (2026) https://BioRender.com/8jpfnct, licensed under CC BY.

The selection process followed PRISMA-ScR recommendations. Records identified through database searching, grey literature, and reference-list screening were evaluated in two stages: title/abstract screening followed by full-text assessment. Screening was performed by the lead author using predefined eligibility criteria. Records with uncertain eligibility were discussed with the co-authors, and final inclusion decisions were made by consensus. Screening was therefore not conducted as blinded independent dual screening; instead, co-author review was used to resolve methodological or technical uncertainty during eligibility assessment and synthesis. Exclusions were applied for domain irrelevance, insufficient methodological detail, duplication, or inaccessibility. The staged counts are reported above and visualized in the PRISMA-ScR flow diagram (Figure 3).

### 3.5 Data charting and appraisal

Data from the included studies (*N* = 87) were systematically charted using a standardized template capturing PET type, biomedical modality, lifecycle stage (training or deployment), privacy parameters, utility metrics, and deployment considerations. Additional fields documented threat-model assumptions, study design, and reported limitations. To maintain transparency and comparability, data extraction was piloted on a subset of studies before being applied across the corpus.

Critical appraisal was conducted using a tailored rubric assessing (i) adequacy of threat models, (ii) clarity of methodological reporting, (iii) reproducibility of experiments, (iv) transparency of privacy–utility trade-offs, and (v) realism of deployment claims. Appraisal findings informed the synthesis and were not used as exclusion criteria. This rubric was designed to move beyond descriptive mapping and provide structured critique of PET applicability in biomedical AI contexts.

## 4 Privacy-enhancing technologies (PETs)

Biomedical AI demands privacy solutions that address the sensitivity and complexity of data such as imaging, genomics, longitudinal records, and multimodal inputs. These datasets encode not only personal but also familial and population-level information. Consequently, PETs in this domain must balance formal privacy guarantees with clinical utility while respecting diverse legal and institutional constraints.

This section reviews core PETs applied in biomedical AI, emphasizing their theoretical bases, implementation trade-offs, and suitability across different stages of the model lifecycle. An evaluation rubric (Table 2) compares principal PET families across six dimensions: privacy strength, utility retention, scalability, computational overhead, fairness impact, and compliance alignment. This framework provides a baseline for the subsections that follow, beginning with differential privacy, a mathematically grounded mechanism for limiting information leakage during model development.

**Table 2:** Evaluation rubric for PETs in biomedical AI.

PET	Privacy	Utility	Scalability	Overhead	Fairness	Compliance
DP	High (*ε*-bounded)	Mod–Low	High	Low	Risk of subgroup harm	Strong
FL	Mod (grad. leakage)	High	High	Mod (comm.)	Non-IID issues	Strong
SMPC	High	High	Low–Mod	Very High	Neutral	Strong
HE	High	High	Low	Extreme	Neutral	Strong
Synthetic Data	Mod–High; release-dependent	Mod; task-dependent	High	Mod	Subgroup fidelity risk	Context-dependent
Hybrid	Very High	Mod	Mod	High	Case-dep.	Strong

Evaluation rubric summarizing core PET families in biomedical AI.

### 4.1 Differential privacy for biomedical AI

Differential privacy (DP) provides a rigorous mathematical framework for protecting individual-level information in machine-learning models. In biomedical AI, where training datasets often contain inherently identifiable features such as genetic profiles, imaging biomarkers, and rare disease phenotypes, DP offers formal guarantees against a wide range of privacy threats. Its core principle ensures that including or excluding a single individual's data does not substantially affect model output, even when adversaries possess auxiliary knowledge (8).

#### 4.1.1 Technical foundations and practical implementations

DP is defined by two parameters: epsilon (*ε*), the privacy budget bounding maximum leakage, and delta (*δ*), the probability that this guarantee may fail. Smaller *ε* values provide stronger privacy but typically degrade performance.

In deep learning, Differentially Private Stochastic Gradient Descent (DP-SGD) limits sensitivity by clipping individual gradients and adding calibrated Gaussian noise to aggregated updates. Frameworks such as Opacus (PyTorch) and TensorFlow Privacy integrate DP-SGD into biomedical pipelines (23, 40).

Recent advances include Rényi Differential Privacy (RDP), which uses Rényi divergence for tighter privacy accounting (41), and privacy amplification by subsampling, which reduces effective leakage through random batch selection (42). DP has also been adapted for generative modeling in biomedical contexts, enabling privacy-preserving synthetic data creation through *Generative Adversarial Networks* (GANs) and *Variational Auto-Encoders* (VAEs) (43). These approaches generate benchmarking or cohort-simulation datasets with formal disclosure-risk guarantees.

In federated learning (FL), DP is typically applied at the client level: each institution perturbs model updates before sharing them. Combined with secure aggregation, this prevents inspection of individual updates (9).

#### 4.1.2 Domain-specific limitations and practical challenges

Despite strong theoretical protection, real-world deployment faces several challenges. The most common is performance degradation, especially in rare-disease prediction or minority-subgroup analysis. Small, imbalanced datasets are highly sensitive to DP noise, which can obscure critical signals and reduce diagnostic reliability (44). A uniform noise distribution can also disproportionately affect underrepresented groups, amplifying algorithmic bias (27).

There is no consensus on acceptable *ε* thresholds. Consumer applications may tolerate *ε* between 5 and 10, whereas biomedical research often targets *ε* ≤ 2 to reflect stricter ethical and legal expectations (45). These values are not universal; adequacy depends on dataset heterogeneity, adversarial strength, and the frequency of output release. Selecting inappropriate *ε* values can weaken protections, particularly under adversaries with strong prior knowledge.

DP may further reduce model interpretability and stability. Injected noise introduces non-deterministic gradients and prediction variability, problematic in clinical workflows requiring consistent and explainable outputs (11). Such constraints are most critical in high-stakes settings such as genomic risk scoring, ICU mortality prediction, and cancer subtype classification, where precision and privacy must be carefully balanced.

#### 4.1.3 Empirical observations and use case boundaries

**Figure 4:**
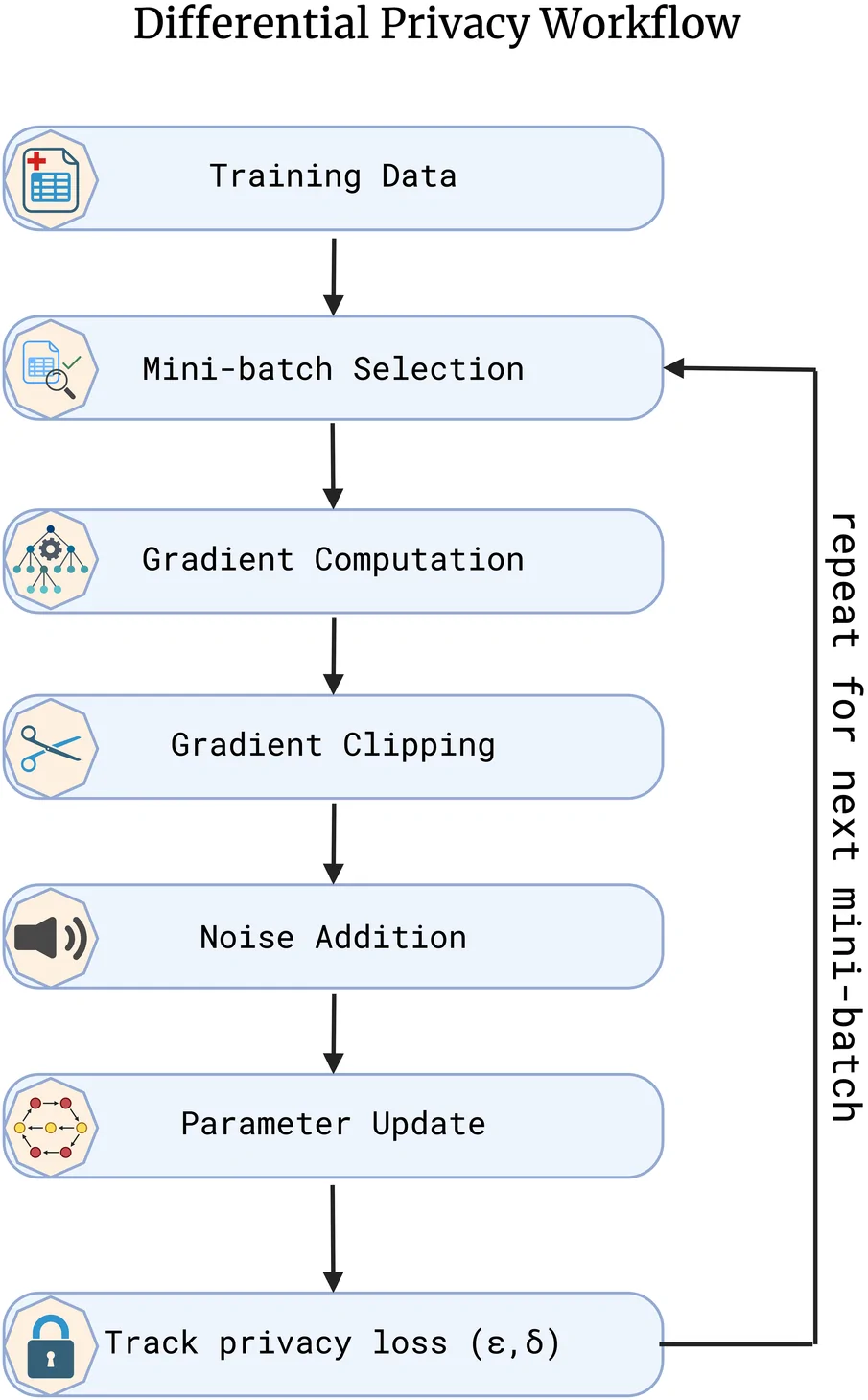
Workflow of differential privacy during model training. Training data is used in mini-batches to compute gradients, which are clipped and perturbed with noise before parameter updates. The cumulative privacy budget (ɛ, *δ*) is tracked across iterations to ensure privacy guarantees. Created in BioRender. Ay, S. (2026) https://BioRender.com/sig2b1n, licensed under CC BY.

The effectiveness of DP depends on dataset complexity, model architecture, and privacy-parameter calibration. Figure 4 illustrates the typical workflow: gradients are clipped, noise is added, and the cumulative budget is tracked.

Shukla et al. demonstrated that a federated DP system for breast-cancer diagnosis achieved 96.1% accuracy at *ε* ≈ 1.9, matching the 96.0% baseline from a centralized non-private model (46). Success depended on balanced data and tuned parameters. Conversely, studies on high-dimensional or imbalanced datasets report 5%–10% performance loss at similar budgets (44). The effect is not uniform; minority-class accuracy can degrade faster under DP constraints (27), underscoring the need for subgroup-aware calibration or hybrid PET designs.

#### 4.1.4 Optimal use cases

DP performs best when formal, quantifiable privacy guarantees are required and centralized pooling is infeasible. It is effective for aggregate-statistics reporting (47), privacy-preserving federated learning, and synthetic-data generation via DP-GANs and DP-VAEs. However, DP is rarely applied during inference in biomedical AI. Adding noise to prediction outputs could extend protection to deployed models, but interpretability and reliability concerns limit adoption (7). Consequently, most biomedical DP implementations focus on training, leaving inference-time vulnerabilities unresolved.

#### 4.1.5 Takeaway

Differential privacy remains a cornerstone PET in biomedical AI, offering strong formal guarantees and proven success in collaborative environments. Its deployment must consider dataset characteristics, subgroup disparities, interpretability needs, and context-appropriate *ε* calibration. Because *ε* is a relative rather than absolute measure, selection should reflect dataset size, adversarial assumptions, and release frequency. In practice, DP achieves optimal performance when combined with complementary PETs that preserve clinical utility. Representative applications are summarized in Table 3, illustrating how dataset type and privacy budgets influence performance trade-offs.

**Table 3:** Representative differential privacy Use cases in biomedical AI.

Use case	Dataset type	Privacy budget (*ε*)	Performance drop	Key insight
Breast cancer diagnosis	Structured (tabular)	∼1.9	∼0.1%	Near-baseline accuracy achievable with tuning
ICU mortality prediction	Multimodal, longitudinal	≤2.0	5–10%	DP noise disrupts temporal dependencies in sequential data
Rare disease Classification	Imbalanced classes	≤2.0	8–12%	Performance degradation is amplified for minority cohorts
Summary statistics publication	Aggregate outputs	≤1.0	Minimal	Query-based noise calibration maintains statistical fidelity

The table illustrates how DP affects utility across representative biomedical tasks, highlighting variability by dataset type, cohort imbalance, and output modality.

### 4.2 Secure multiparty computation (SMPC)

Secure multiparty computation (SMPC) is a cryptographic protocol that allows multiple parties to jointly compute a function over their private inputs without revealing those inputs. In biomedical AI, where patient data are siloed across hospitals and research institutions, SMPC enables collaborative model training and analytics without sharing raw patient records (25).

#### 4.2.1 Technical foundations

**Figure 5:**
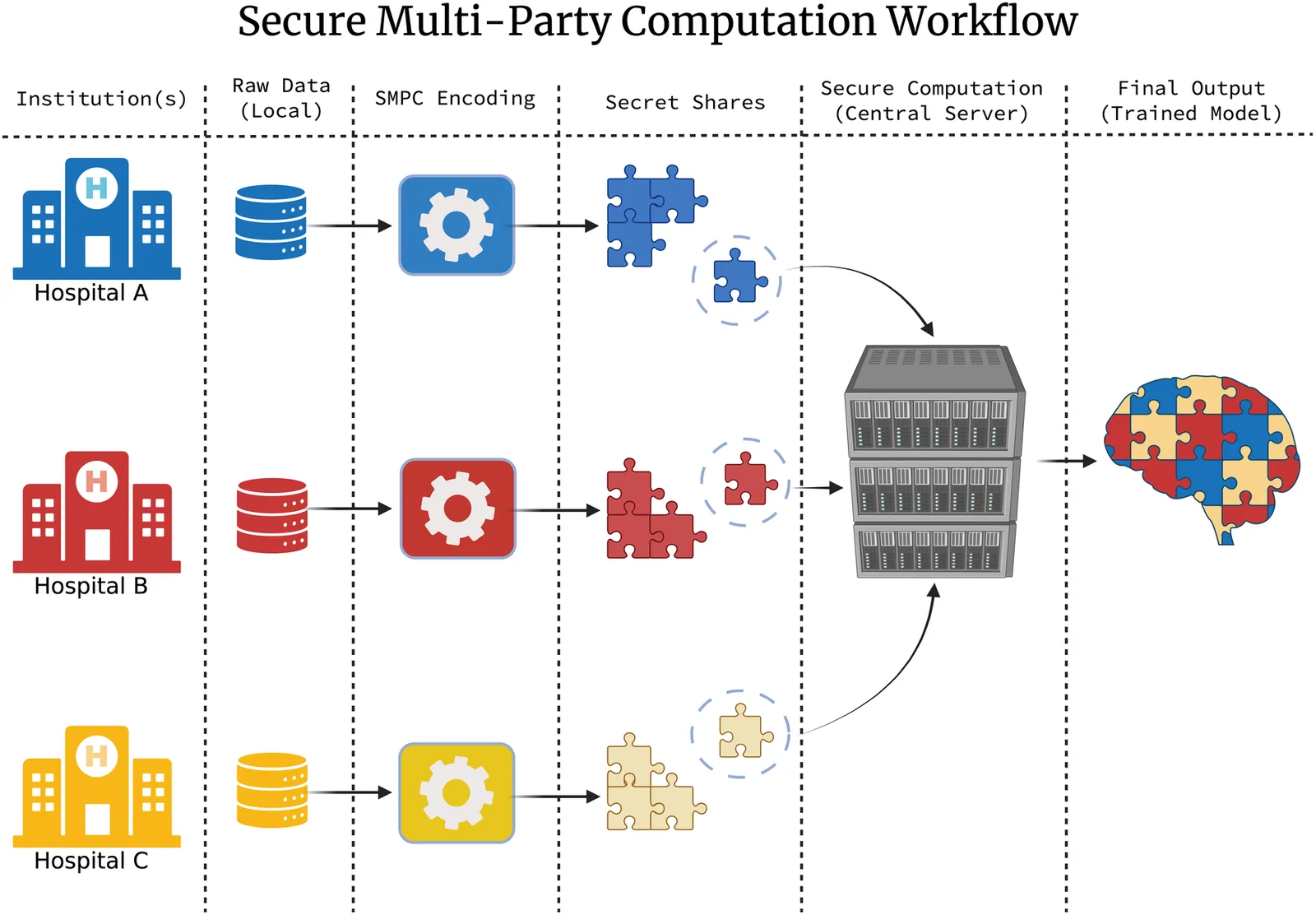
Workflow of secure multi-party computation (SMPC). Each hospital locally transforms its raw data into secret shares, which are distributed to a central computation server. The server performs joint computation on the shares without accessing raw data, producing a privacy-preserving trained model. Created in BioRender. Ay, S. (2026) https://BioRender.com/rn9wjn1, licensed under CC BY.

SMPC is based on *secret sharing*, where each input is split into randomized “shares” distributed among participants. Computations occur collectively on these shares in a privacy-preserving manner, and only the final output is reconstructed after all parties complete their roles. No individual input is ever exposed (25). Figure 5 illustrates the workflow: data are divided into shares, jointly computed, and reconstructed only as final outputs.

Several well-established protocols support SMPC. Arithmetic secret sharing is used for addition and multiplication; Yao's garbled circuits enable efficient two-party function evaluation; and threshold homomorphic encryption aggregates encrypted data across participants without decryption. Frameworks such as CrypTen integrate these methods into machine-learning pipelines through PyTorch-compatible APIs for secure tensor operations. Systematization studies note that most tools assume a *semi-honest* threat model, where parties follow the protocol but may attempt to infer intermediate information (48, 49). These frameworks now support GPU-accelerated model training for image and text tasks.

#### 4.2.2 Privacy advantages during model training

SMPC provides strong privacy during model development because no participant accesses another's raw data or intermediate results. Under semi-honest or passive threat models, reconstruction of private inputs is infeasible without collusion. Unlike differential privacy, SMPC avoids noise injection, preserving fidelity and enabling exact computation under correct protocol execution. However, these guarantees hold only within the assumed threat model and do not extend to malicious or colluding participants (24). This feature is valuable in high-stakes biomedical contexts such as genomic risk prediction or diagnostic classification, where even small performance losses can have clinical consequences.

#### 4.2.3 Practical limitations and constraints

Despite its strengths, SMPC faces challenges that limit its scalability and usability in biomedical deployments. The foremost issue is **computational overhead**: secure tensor operations such as matrix multiplication or nonlinear activation are significantly slower than their plaintext equivalents. Reported slowdowns range from 10× to more than 100× in deep learning applications (24, 49).

**Communication latency** is another major bottleneck. Many SMPC protocols require multiple interaction rounds per operation, generating substantial coordination overhead, especially in geographically distributed hospital networks (25). Current frameworks are also optimized for a small number of participants; as parties or data dimensionality increase, systems like MP-SPDZ or CrypTen struggle to maintain performance and synchronization.

Importantly, SMPC protects data only during the collaborative computation phase. Once training is complete, the resulting model is no longer covered and becomes vulnerable to inference-time attacks unless combined with complementary PETs (25). This lifecycle limitation underscores the need for **hybrid approaches**, pairing SMPC with differential privacy for training-time protection or with homomorphic encryption and trusted execution environments (TEEs) for inference (50).

#### 4.2.4 Deployment scenarios and use cases

SMPC is particularly suited for biomedical AI scenarios governed by strict data-sharing restrictions or limited institutional trust. Common applications include international research partnerships, rare-disease registries, and multi-center trials involving sensitive domains such as mental health or genomics.

Ballhausen et al. implemented a GDPR-compliant SMPC system enabling collaborative analysis of rare-tumor data across European centers without exposing patient-level records (51). Kamm et al. demonstrated large-scale genome-wide association studies across biobanks with full privacy preservation (52), while Tkachenko et al. later extended this approach to hundreds of institutions with significant efficiency gains (53).

Although SMPC provides strong training-time protection, it is not a standalone solution. Trained models remain exposed to post-deployment inference attacks, necessitating integration within **hybrid PET pipelines**. In practice, SMPC is combined with differential privacy to bound training-time leakage or with homomorphic encryption and TEEs to secure inference (54). Such layered architectures ensure broader coverage across the model lifecycle and align with regulatory expectations for end-to-end safeguards in biomedical AI.

### 4.3 Homomorphic encryption (HE)

**Figure 6:**
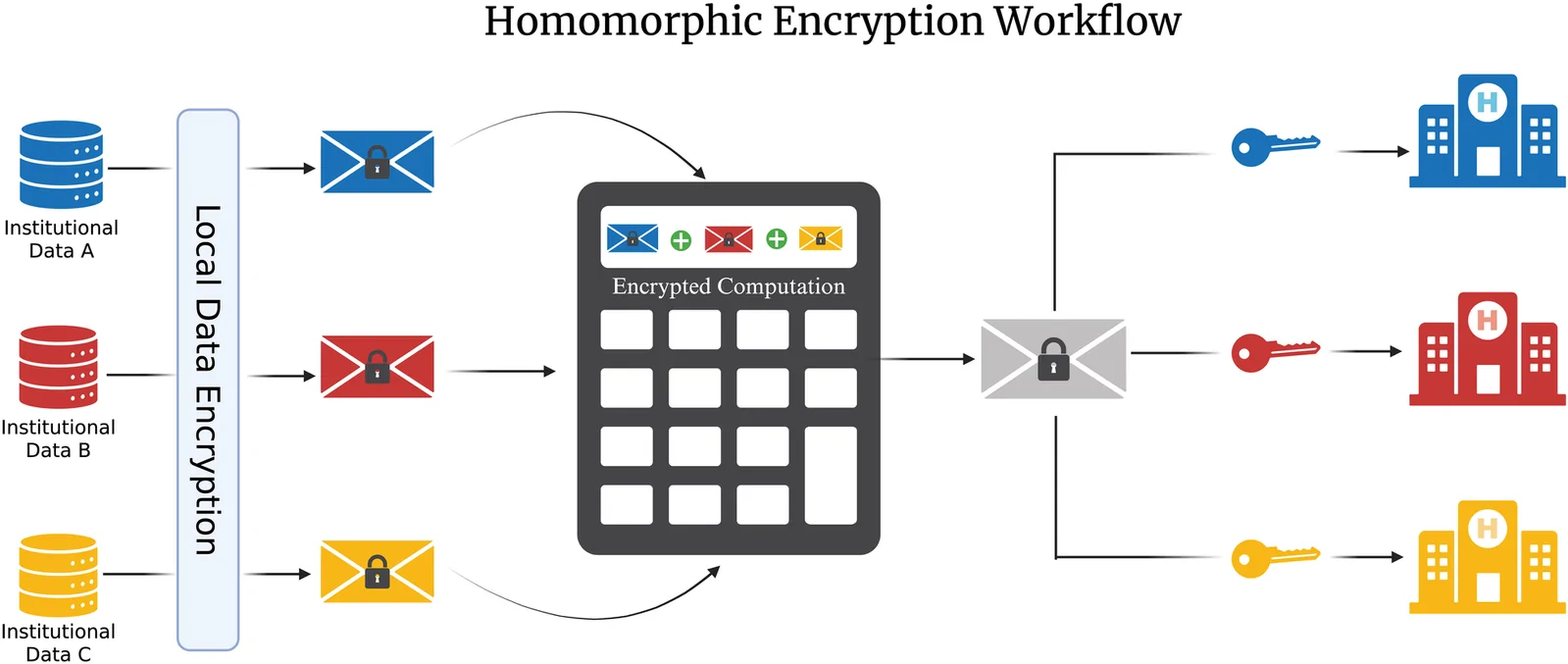
Workflow of homomorphic encryption (HE). Each institution encrypts its local dataset before transmission, ensuring no raw data leaves its site. Encrypted values are sent to a central computation server, which performs mathematical operations directly on ciphertexts without decryption. The aggregated encrypted result is then returned to participating hospitals, where each institution uses its secret key to decrypt and obtain the final outcome. Created in BioRender. Ay, S. (2026) https://BioRender.com/qplpbck, licensed under CC BY.

Homomorphic encryption (HE) enables computation directly on encrypted data so that, after decryption, the result matches what would have been obtained on plaintext. Figure 6 illustrates this workflow: encrypted patient data are processed to produce encrypted outputs, which only authorized parties can decrypt. This property makes HE well suited for biomedical AI applications in which either model parameters or input data must remain confidential throughout inference.

#### 4.3.1 Technical foundations

HE schemes differ by computational expressiveness. *Partially homomorphic encryption* (PHE) supports a single algebraic operation, such as addition (Paillier) or multiplication (RSA). *Somewhat homomorphic encryption* (SHE) permits a limited number of both operations but is constrained by noise growth and circuit depth. *Fully homomorphic encryption* (FHE), the most powerful class, supports arbitrary computation on ciphertexts.

Modern FHE protocols rely on lattice-based cryptography, including BGV *(Brakerski-Gentry-Vaikuntanathan)* and CKKS *(Cheon-Kim-Kim-Song)*, the latter enabling approximate arithmetic over real numbers and therefore well suited to biomedical data (31, 55). These have been implemented in libraries such as Microsoft SEAL, PALISADE, and TenSEAL, which support secure matrix operations and encrypted neural-network inference. Current implementations remain optimized for inference rather than training due to computational complexity (56).

#### 4.3.2 Biomedical applications and privacy advantages

HE is most effective for **secure inference**, such as privacy-preserving diagnostics and genomic analytics. A healthcare provider can encrypt patient data before transmitting them to a remote AI service, which performs computation on the ciphertexts and returns encrypted predictions, ensuring the provider never sees raw data. Likewise, encrypted models can be deployed to protect intellectual property from reverse-engineering.

In genomics, HE has supported secure genome-wide association studies (GWAS) without revealing individual variants (32, 57) and privacy-preserving queries over genomic databases through protocols such as SIG-DB (58, 59). Recent work extends these methods to multiparty HE frameworks that enable collaborative precision-medicine analytics across institutions without raw-data exchange (60). Collectively, these advances illustrate a “zero-trust” computing paradigm in which both model and input remain cryptographically protected.

#### 4.3.3 Limitations in biomedical deployment

Despite strong security, HE faces several practical constraints. **Computation cost** is the major obstacle: fully homomorphic operations are typically 10×–1,000× slower than plaintext inference, depending on circuit depth and parameters (61). This limits use to latency-tolerant environments or those with specialized hardware acceleration. Training under HE remains largely impractical because encrypted gradient updates and back-propagation impose prohibitive memory and time costs; thus, HE is primarily used for inference.

**Circuit-depth constraints** present another challenge. Many biomedical models depend on nonlinear functions such as ReLU or SoftMax, which are not natively supported under HE and must be approximated by low-degree polynomials, often reducing accuracy. Although research on encrypted activation approximations continues (62), performance penalties remain. Existing libraries also lack efficient GPU acceleration, further limiting scalability for high-throughput clinical workflows.

#### 4.3.4 Deployment scenarios and interoperability

Despite inefficiencies, HE is valuable where privacy must be absolute. It is well suited for secure prediction services on cloud infrastructure, enabling institutions to outsource computation without exposing raw patient data. HE also facilitates cross-institutional collaborations involving sensitive clinical or genomic inputs that must remain under local governance.

In such deployments, HE is rarely used alone. It is commonly combined with other PETs, for example, paired with SMPC to distribute trust among parties or with differential privacy (DP) to bound cumulative disclosure risks (54). These hybrid approaches enhance protection across both training and inference phases.

In summary, homomorphic encryption provides a robust cryptographic foundation for privacy-preserving inference in biomedical AI. While its high computational cost and limited hardware support constrain widespread deployment, continued optimization and hybrid integration make HE one of the most promising paths toward secure computation in clinical and research environments.

### 4.4 Federated learning (FL)

Federated learning (FL) is a decentralized paradigm that enables collaborative model training across institutions without transferring raw data. This design suits biomedical AI, where privacy regulations and ethics often prohibit sharing identifiable patient information. By training models locally and exchanging only parameter updates, FL provides a scalable framework for privacy-preserving learning across hospitals, biobanks, and research centers.

#### 4.4.1 Technical overview and implementation strategies

In a typical workflow, each institution trains a model locally and sends weight updates or gradients to a coordinating server, which aggregates them to refine a global model and redistributes it for further training. This iterative process continues until convergence (63).

Several algorithms address the challenges of heterogeneous biomedical data. *Federated Averaging* (FedAvg) remains the baseline, but its performance degrades on non-identically distributed (non-i.i.d.) data. *FedProx* stabilizes training through proximal terms, while optimizers such as *FedAdam* and *FedYogi* adapt centralized momentum methods to federated settings. Frameworks including TensorFlow Federated, PySyft, Flower, NVIDIA Clara, and NVFlare operationalize these approaches for biomedical use (64, 65). Robust deployment, however, requires orchestration layers for authentication, logging, and fault tolerance (66).

More recent FL research has moved beyond baseline aggregation and optimizer choices toward methods that explicitly address client drift, non-i.i.d. data, unstable participation, and adversarial updates. These include personalized or clustered FL strategies that adapt models to site-specific distributions, drift-correction and adaptive weighting methods that reduce instability across heterogeneous clients, and robust aggregation or anomaly-detection frameworks designed to limit the impact of poisoned or backdoored updates (28, 64, 66, 67). In biomedical settings, these advances are particularly relevant because institutions often differ in patient mix, imaging protocols, coding practices, hardware capacity, and network reliability. However, many of these methods still require careful validation under realistic clinical workflows before they can be treated as mature deployment standards.

#### 4.4.2 Biomedical privacy advantages

FL aligns naturally with healthcare privacy goals because patient data never leave institutional boundaries. This satisfies data-residency rules and confidentiality mandates, mitigating centralized-breach risks and supporting compliance with regulations such as GDPR and HIPAA. By enabling collaboration across diverse institutions, FL improves model generalizability through exposure to varied populations and clinical practices.

FL has been applied to cross-hospital ICU mortality prediction, distributed rare-disease modeling, and clinical NLP. Sheller et al. demonstrated that federated deep learning enabled accurate brain-tumor segmentation across multiple hospitals without central data pooling, validating its clinical feasibility (68).

#### 4.4.3 Privacy limitations and vulnerabilities

Despite decentralization, FL alone does not guarantee privacy. Model updates may leak sensitive information through gradients or model behavior. Gradient-inversion attacks can reconstruct patient inputs from shared updates, especially with small batch sizes or unprotected transmissions (35, 36). Membership inference can test whether specific patients contributed to training (7), and property inference may reveal cohort-level trends such as disease prevalence (22). FL is also exposed to poisoning attacks, where malicious clients upload corrupted updates to bias model behavior, critical in diagnostic or decision-support systems.

To mitigate these risks, FL is often combined with other PETs. Differential privacy (DP) adds calibrated noise to local updates, masking individual contributions. Secure aggregation prevents servers from viewing individual updates, and homomorphic encryption (HE) can encrypt transmitted parameters, though with computational cost. Hybrid stacks combining FL + DP + secure aggregation are now standard in biomedical deployments, balancing privacy and utility (54).

#### 4.4.4 Deployment and operational challenges

Practical deployment of FL in biomedical contexts is challenged by systemic and architectural issues. Non-i.i.d. data, which is common in healthcare due to differing patient populations, sensors, and even documentation styles, can lead to unstable convergence and reduced generalization. Hospital systems may also experience variable participation due to intermittent network connectivity, administrative constraints, or hardware limitations. Additionally, high-resolution biomedical data, such as imaging or genomic sequences, can saturate communication channels, introducing latency and scaling issues.

Another persistent challenge lies in quality control. Since model updates are computed locally, validating their correctness, fairness, or contribution becomes difficult. Without centralized access to raw data, ensuring auditability and interpretability of updates requires additional governance structures or trusted hardware components. Recent studies suggest using blockchain-based audit trails or trusted execution environments (TEEs) to enhance accountability and transparency in federated biomedical networks (69).

Various mitigation techniques have been proposed, including gradient sparsification to reduce communication bandwidth, client sampling to balance training fairness, and local validation to detect unproductive updates. Nevertheless, FL deployments in biomedical settings must remain highly tailored to local infrastructure, population characteristics, and governance models.

#### 4.4.5 When FL is most effective

Federated learning is most appropriate when data cannot be centralized for legal, ethical, or logistical reasons but collaborative training remains desirable. It is well suited to multi-institutional research consortia, geographically distributed disease registries, and mobile-health monitoring platforms. FL also supports personalized or adaptive models where inference must reflect local data distributions without violating privacy.

**Figure 7:**
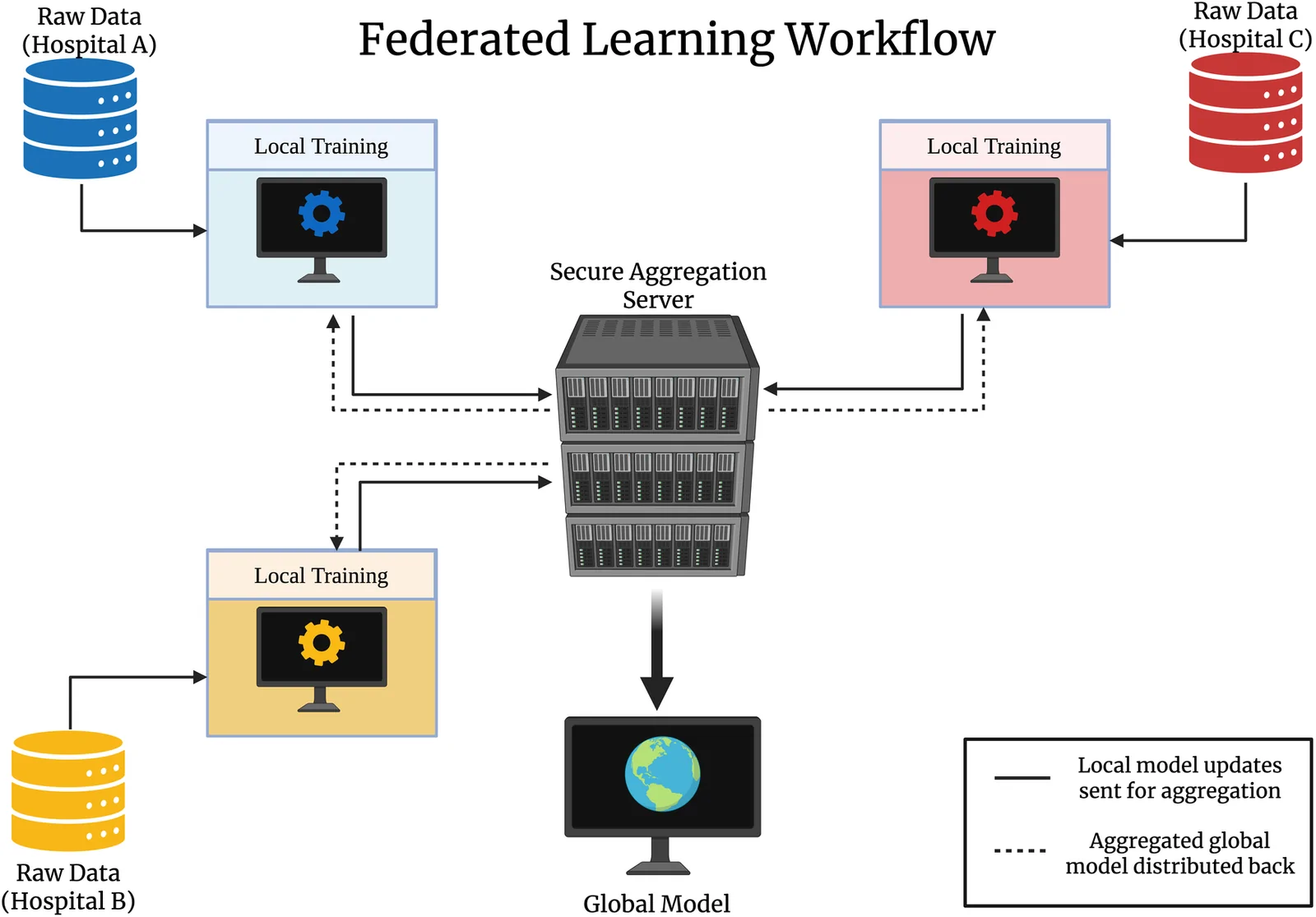
Federated learning workflow. Local institutions train models on their own data and send only model updates (solid arrows) to a central aggregation server. The server combines these updates and redistributes the aggregated model parameters back to the institutions (dashed arrows), ensuring that raw data never leaves the local environment. Created in BioRender. Ay, S. (2026) https://BioRender.com/6nvxn6k, licensed under CC BY.

However, FL should not be viewed as a complete privacy solution. It is most powerful when integrated with complementary PETs that secure communication, obscure individual contributions, and extend protection into post-training phases. Figure 7 illustrates the federated-learning workflow, where institutions train models locally and share only aggregated updates with a coordinating server.

### 4.5 Synthetic data generation

Synthetic data generation is increasingly used as a privacy-enhancing strategy for biomedical AI because it can support data sharing, benchmarking, and model development without directly releasing original patient records. Rather than distributing source clinical, imaging, genomic, or wearable data, a generative process learns statistical patterns from the original dataset and produces new records intended to preserve analytic utility while reducing direct identifiability. This approach is attractive when institutions need to enable downstream analysis, external validation, or early model development but cannot share patient-level data because of regulatory, contractual, or governance constraints (10).

Synthetic data, however, should not be treated as automatically anonymous or risk-free. Its privacy value depends on the generation method, the release setting, the similarity between synthetic and source records, and the adversary's auxiliary knowledge. If the generator overfits or preserves rare patterns too closely, synthetic records may still create residual risks of membership inference, attribute inference, or linkage to external information (17, 18). These risks are especially relevant in biomedical settings where rare diseases, small subgroups, and longitudinal records may contain highly distinctive patterns.

Differential privacy can strengthen synthetic data generation by limiting memorization during model training. For example, differentially private GANs apply DP mechanisms during generative modeling to reduce the influence of individual training records (43). Even with such safeguards, synthetic data generation remains a complementary PET rather than a substitute for disclosure-risk testing, access control, and governance. Responsible use requires explicit privacy evaluation, subgroup-level utility assessment, and clear limits on downstream reuse.

### 4.6 Hybrid and emerging PET combinations

No single privacy-enhancing technology (PET) fully satisfies the combined requirements of privacy, utility, and regulatory compliance in biomedical AI. Hybrid architectures address this limitation by combining complementary PETs: differential privacy (DP) for statistical protection, secure multiparty computation (SMPC) or homomorphic encryption (HE) for cryptographic safeguards, and federated learning (FL) for data locality. Together, these methods defend against training- and inference-stage threats while balancing formal guarantees with clinically acceptable performance. DP provides quantifiable privacy but may degrade accuracy on small or imbalanced datasets; SMPC and HE deliver strong cryptographic protection at the cost of efficiency; FL preserves data residency but exposes model updates unless paired with secure aggregation or local DP (9). Table 4 compares major PETs by strengths, limitations, scalability, and representative biomedical uses.

**Table 4:** Comparative overview of major PETs in biomedical AI.

PET(s)	Strengths	Limitations	Primary use	Scalability/deployment	Use case
Differential privacy (DP)	Provable privacy guarantees; widely applicable	Utility loss; especially on small or imbalanced datasets	Training and inference	Scales well in software; minimal infra overhead, but parameter tuning critical	Model training under legal/ethical constraints (23, 45)
Secure multi-party computation (SMPC)	No raw data exposure; strong training-phase protection	High computation & communication overhead	Training	Limited scalability beyond small consortia due to quadratic communication costs	Cross-institutional training without central aggregator (25, 49)
Homomorphic encryption (HE)	Computations over encrypted data; inference-time privacy	Extremely slow; impractical for complex models	Inference	Poor scalability for deep learning; feasible for shallow or linear models	Encrypted inference for genomics or diagnostics (31, 57)
Federated learning (FL)	Raw data stays local; scalable to multiple sites	Gradient leakage risk; requires additional PETs	Training	High scalability to 100s–1,000s of nodes, but bandwidth heterogeneity is a bottleneck	ICU mortality prediction, rare disease modeling (63)
FL + DP + SECURE AGGREGATION	Formal privacy + server-side obfuscation	Performance trade-offs; calibration complexity	Training	Scales better than SMPC but limited by secure aggregation protocols	COVID-19 modeling, diabetic retinopathy (54)
FedML-HE	Encrypted federated updates	Encryption latency	Training/aggregation	Limited by HE overhead	Privacy-preserving FL aggregation (71)
FAMHE	End-to-end encrypted analytics with near-centralized utility	High communication and encryption overhead	Training	Scales to mid-sized consortia; overhead grows linearly with institutions	Secure federated biomedical analytics across biobanks (60)
DP + Generative modelling	Synthetic data with formal privacy guarantees	Limited realism; not usable for all modalities	Training (data generation)	Scalable for dataset release; utility depends on generative model capacity	Sharing privacy-safe biomedical datasets (43)

#### 4.6.1 Common hybrid configurations

Hybrid PETs are increasingly used because no single method can satisfy utility, scalability, and regulatory constraints at once. In practice, many systems combine FL with cryptographic or statistical safeguards to extend protection across institutions while maintaining accuracy.

A common design integrates FL + local DP + secure aggregation, applied in multi-institutional imaging tasks such as diabetic-retinopathy classification and COVID-19 severity prediction (54). DP noise obscures individual contributions, while secure aggregation prevents server-side inspection of institutional updates. These systems achieve near-baseline diagnostic accuracy, showing that privacy can be strengthened without major performance loss (70).

Another configuration merges FL + HE, exemplified by FedML-HE (71), where encrypted model updates protect training-time aggregation and reduce trust in central servers. Although slower than plaintext FL, such schemes enable collaboration when institutional confidentiality is paramount. Multiparty HE systems such as FAMHE (60) securely aggregate encrypted biomedical data and have achieved near-centralized performance in genome-wide association studies (52).

Hybridization also extends beyond FL. HE–DP pipelines maintain ciphertext protection during inference while adding DP noise at the output layer to limit reconstruction risk. Generative models trained with DP [for example, DP-GANs (43)], produce synthetic datasets that mimic real cohorts for benchmarking and sharing without exposing patient records.

Together, these approaches illustrate the complementary nature of PETs: DP provides formal guarantees, cryptographic tools prevent direct exposure, and FL ensures local data retention. Layering them enhances resilience against diverse attack vectors and reduces dependence on any single assumption.

Hybrid frameworks are particularly useful in cross-jurisdictional research, rare-disease modeling with small cohorts, and generative-data applications where inversion risks are high. Current trends emphasize modular PET stacks, hardware-accelerated cryptography, and integrated privacy-budget management that spans multiple PETs (56). These efforts aim for scalable, composable privacy solutions adaptable to evolving biomedical demands.

#### 4.6.2 Limitations and composability challenges

Despite their promise, hybrid PETs face notable obstacles. The foremost is composability of privacy guarantees. When PETs are applied sequentially, their assumptions may misalign. For example, adding DP noise to outputs from an HE-protected pipeline can distort calibration and weaken guarantees. No standardized framework yet exists for evaluating cumulative security in multi-PET pipelines, which complicates claims of end-to-end protection (67).

Even when privacy holds, hybridization introduces performance costs. Encryption, aggregation, and repeated noise addition increase latency, communication load, and energy use, especially for high-resolution imaging or genomic tasks. These trade-offs affect both efficiency and the sustainability of PET adoption at scale.

Benchmarking represents another persistent gap. Current evaluations are fragmented across studies and rarely use unified protocols that capture privacy, utility, fairness, and computational cost in an integrated way (65). This makes it difficult to compare competing designs or to establish reliable baselines for clinical deployment.

Finally, regulatory uncertainty persists. Agencies have not issued explicit criteria for combined PETs under GDPR or HIPAA. Institutions therefore depend on case-by-case legal interpretation, complicating cross-border collaborations and slowing the adoption of technically sound solutions.

#### 4.6.3 Emerging PETs

Several emerging approaches are expanding the PET landscape and may soon influence biomedical AI practice.

Trusted execution environments (TEEs) provide hardware-based isolation for sensitive computation. By executing operations within secure enclaves, TEEs can protect orchestration layers such as aggregators or schedulers, or support end-to-end training with remote attestation. Recent prototypes extend enclave capabilities to AI accelerators and can reduce the trusted computing base. However, they remain vulnerable to attestation failures and side-channel attacks that must be mitigated before deployment in regulated biomedical workflows (26, 72).

Zero-knowledge proofs (ZKPs) strengthen auditability through verifiable computation. In verifiable machine learning, ZKPs allow one party to prove that training or inference was performed correctly without revealing inputs or model internals. New protocols reduce prover cost for nonlinear layers such as ReLU and GELU and now support proofs-of-training for deep networks. Although end-to-end demonstrations in clinical contexts are scarce, ZKPs suggest a path toward auditable biomedical pipelines (73, 74).

Efforts are also under way to adapt large models to privacy-preserving workflows. Federated fine-tuning of transformers or large-language models (LLMs), combined with secure aggregation and optional client-side DP, achieves accuracy near centralized baselines. Biomedical NLP studies show that such methods enable collaborative text mining without exposing raw notes, making them promising for multi-institution deployments (75, 76).

Finally, differentially private diffusion models (DPDMs) are emerging as next-generation generative PETs. These models produce high-fidelity synthetic biomedical data while maintaining formal privacy guarantees. Early results in TMLR indicate that DP training and latent-diffusion fine-tuning can preserve downstream utility, though subgroup fidelity and fairness remain open challenges.

Taken together, these emerging PETs point to a transition toward composable privacy architectures. Robust protection in biomedical AI will increasingly depend not on isolated mechanisms, but on integrated stacks that combine hardware, cryptography, and statistical safeguards, and are validated under realistic biomedical workloads.

## 5 Infrastructure and deployment models

The deployment of privacy-enhancing technologies (PETs) in biomedical AI requires careful attention to both infrastructure and operational conditions. Unlike algorithmic design (Sec. 4), deployment involves the physical and digital environments where PETs operate. This section outlines the foundational requirements for reliable and scalable PET adoption in clinical settings, organized into four layers: hardware and computational infrastructure, software frameworks and toolchains, cloud and edge deployment scenarios, and operational challenges including monitoring, cost, and maintenance. Different PET families (e.g., DP, SMPC, HE, FL) impose distinct demands on these layers, which are closely tied to regulatory compliance since technical feasibility often determines whether PET implementations satisfy legal and ethical standards.

### 5.1 Hardware and computational infrastructure

Effective PET deployment depends heavily on suitable hardware. PETs such as fully homomorphic encryption (FHE) and secure multiparty computation (SMPC) are computationally intensive, often requiring several orders of magnitude more resources than plaintext machine learning. Even with hardware acceleration, FHE operations remain 10× to 1,000× slower than non-private equivalents, and cryptographic primitives impose high memory and compute requirements (61)*.* FPGA-based implementations have improved performance; for example, the FAST architecture achieves about 100× faster bootstrapping than CPUs, significantly reducing latency (77). Nevertheless, computational demands remain a bottleneck for high-dimensional biomedical models.

Federated learning (FL) shifts computation to distributed sites, reducing central processing needs but adding communication overhead. High-throughput, stable networks are essential for synchronizing model updates across hospitals (9)*.* Network heterogeneity and latency exacerbate challenges, particularly when handling large imaging or genomic data. SMPC workflows add further complexity since multi-round protocols require frequent message exchanges between sites. These factors underscore the importance of resilient and dedicated networking infrastructure to maintain scalability and reliability in decentralized PET deployments.

Energy consumption also plays a key role. Cryptographic operations in FHE and SMPC often translate to higher power usage compared to traditional AI workloads, which raises concerns about sustainability and operational costs in healthcare environments (56)*.*

Finally, data interoperability remains vital. Standards such as FHIR, HL7, and DICOM enable structured data exchange across systems and help avoid computational bottlenecks caused by inconsistent or *ad hoc* preprocessing (78)*.*

### 5.2 Software frameworks and deployment toolchains

Translating PETs from theory into practice depends on robust software frameworks that encapsulate complex cryptographic primitives while providing accessible interfaces for biomedical researchers. The selected framework strongly influences development effort, reproducibility, and the preservation of formal privacy guarantees in production environments.

In SMPC, libraries such as CrypTen and MP-SPDZ express secure tensor operations in a Pythonic style compatible with PyTorch or NumPy. These tools abstract cryptographic complexity and allow non-specialists to build secure workflows while maintaining rigorous protection (49). For HE, toolkits like Microsoft SEAL, PALISADE, and TenSEAL offer polynomial and matrix operations on ciphertexts, with CKKS-based schemes optimized for real-number computations (31).

For FL, frameworks such as TensorFlow Federated (TFF) and Flower are widely adopted for their modularity and support for heterogeneous client devices. They orchestrate distributed training, secure aggregation, and optional DP integration (9). Built-in simulation environments allow evaluation of performance under variable network and data conditions before live deployment, critical for multi-site biomedical studies.

Containerization and orchestration are key to scaling PET-enabled workflows. Docker and Kubernetes encapsulate PET frameworks with their dependencies, ensuring reproducibility and portability across institutions with different infrastructures. Many biomedical collaborations integrate these containers with continuous integration and deployment (CI/CD) pipelines that automate security patching, cryptographic library updates, and compliance monitoring across sites (79).

Interoperability planning is essential. Most frameworks support standardized data formats such as DICOM for imaging and HL7 FHIR for structured records, reducing schema mismatches and preprocessing effort. Combined with version-controlled model repositories and automated validation workflows, these standards improve operational efficiency and ensure compliance.

### 5.3 Cloud and edge deployment scenarios

Choosing between cloud-based and edge-based architectures requires balancing computational power, latency, and regulatory compliance while preserving privacy assurances.

Cloud infrastructure offers scalable compute resources ideal for demanding PETs such as HE and SMPC. High-performance hardware including GPUs, TPUs, and FPGAs accelerates PET-enabled training and inference on large datasets. For instance, Riazi et al. showed that specialized FPGA designs (HEAX) can accelerate FHE by 164–268× over CPU implementations (80). Cloud ecosystems also include compliance frameworks aligned with HIPAA and GDPR, allowing institutions to inherit pre-certified security controls in collaborative research.

However, full reliance on cloud services increases trust requirements and may raise data-jurisdiction concerns. Cloud providers often replicate or process data across borders, creating potential conflicts with GDPR's cross-border transfer restrictions. This is especially critical for international biomedical collaborations involving sensitive clinical data.

Edge deployment offers a complementary model by processing data locally within hospital networks or near clinical devices. This architecture reduces latency, limits data transmission risk, and supports real-time clinical use. Reviews show that edge computing can improve efficiency by minimizing the data sent to centralized servers while maintaining performance for imaging and patient-monitoring tasks (81). Localized computation also simplifies compliance with data residency laws since data remain within institutional boundaries.

Hybrid architectures combine the two approaches to balance capacity and privacy. Lightweight tasks such as feature extraction or encryption occur at the edge, while compute-heavy workloads like encrypted aggregation or DP accounting are offloaded to the cloud. This structure fits well with federated learning, where local models train on-site and only aggregated updates are sent to central servers (9).

Selecting the optimal strategy depends on data sensitivity, computational needs, latency tolerance, and jurisdictional rules. Flexible hybrid deployments that dynamically distribute workloads across edge and cloud layers are increasingly seen as essential for scaling PET-enabled biomedical AI.

### 5.4 Operational challenges and maintenance

Deploying privacy-enhancing technologies (PETs) in biomedical AI is an ongoing operational process that requires continuous monitoring, governance, and adaptation. Clinical environments are dynamic, and PET configurations must evolve alongside changes in data characteristics, user needs, and regulatory landscapes.

A key technical challenge is managing data drift, which refers to shifts in the statistical properties of biomedical data over time. New diagnostic assays, updates in imaging protocols, or demographic changes within patient populations can erode the validity of previously calibrated PET parameters and reduce both privacy and utility. Addressing this issue requires mechanisms for continuous validation, including privacy-aware model retraining and recalibration routines that evaluate both accuracy and privacy leakage across deployment cycles (82).

Integration with legacy clinical infrastructure is another major obstacle. Many healthcare institutions still operate electronic health record (EHR) systems or imaging archives that were not built for cryptographic computation or differential privacy auditing. Bridging PET workflows with such systems often demands middleware components, interface adapters, or phased implementation strategies to avoid disrupting mission-critical operations (3).

Resource constraints further complicate PET adoption. Homomorphic encryption (HE) and secure multiparty computation (SMPC) impose high computational and memory requirements, which are challenging for smaller hospitals or research centers. Cloud-based augmentation can help alleviate some of these burdens but introduces additional governance and trust concerns as well as cross-border compliance issues (9) (see Sec. 5.3).

Human factors are equally critical for sustainable PET operations. Effective deployment depends on interdisciplinary teams that include IT specialists, data scientists, clinicians, and compliance officers. Without adequate training and oversight, misconfigured PET parameters or outdated cryptographic libraries can compromise privacy. Establishing dedicated PET governance structures within institutions helps mitigate these risks and ensures accountability (83).

Finally, PET maintenance must remain aligned with evolving legal and ethical standards. Regulations such as HIPAA and GDPR continue to change, and the legality or adequacy of certain PET configurations may vary over time. Sustainable governance strategies, including periodic legal reviews, comprehensive audits, and adaptive policy updates, are essential to ensure that biomedical AI systems remain compliant, transparent, and trustworthy in the long term (83).

### 5.5 Case studies of PET deployment in biomedical AI

While privacy-enhancing technologies (PETs) are supported by strong theoretical foundations, their translation into biomedical AI requires careful consideration of practical constraints, institutional governance, and real-world performance. To illustrate these dynamics, we highlight two representative case studies. The first examines federated learning (FL) for ICU mortality prediction on MIMIC-IV, demonstrating how statistical performance can be preserved while adhering to data-locality requirements in critical care. The second analyzes Secure Federated GWAS (SF-GWAS), a hybrid framework combining homomorphic encryption (HE) and secure multiparty computation (SMPC) for large-scale genomic association studies, showing how composable cryptography can scale to biobank-level cohorts. Together, these studies exemplify the opportunities and limitations of PET deployment across distinct biomedical domains, from time-sensitive clinical prediction to population-scale genomics.

#### 5.5.1 Vieira et al.: Federated Learning for ICU Mortality Prediction on MIMIC-IV

##### 5.5.1.1 Clinical context and objective

Accurate ICU mortality prediction informs triage, resource allocation, and early intervention. While large datasets like MIMIC-IV are widely used for benchmarking, most prior approaches assume centralized pooling, which is often infeasible due to privacy and governance restrictions. Vieira et al. (84) investigated whether federated learning (FL) could deliver predictive performance comparable to centralized models on MIMIC-IV, while ensuring that no raw patient data left institutional boundaries.

##### 5.5.1.2 Data and governance

The study leveraged MIMIC-IV (85), a publicly available, de-identified critical-care database covering more than 380,000 ICU stays and over 250,000 patients. The cohort for this analysis was derived from structured clinical variables (demographics, vitals, labs) collected within the first 24 h of admission, with in-hospital mortality as the outcome. For simulation, the dataset was partitioned into institutional “clients,” each holding a subset of patient records. This design mimicked federated environments where sites contribute locally trained models but retain full custody of their data.

##### 5.5.1.3 Threat model

The deployment assumed an honest-but-curious server: model updates were shared with a central aggregator that performed federated averaging (FedAvg) but was not permitted to inspect raw records. While this protected against direct data exposure, the study did not incorporate additional PET layers such as differential privacy or secure aggregation, leaving updates theoretically vulnerable to gradient-based inference attacks.

##### 5.5.1.4 PET stack and rationale

The PET stack consisted of FL with FedAvg aggregation. Local institutions trained models on their partitions and transmitted only parameter updates to the server. The choice of FedAvg was motivated by its simplicity, established stability across diverse client configurations, and prior adoption in healthcare FL benchmarks.

##### 5.5.1.5 System architecture

A hub-and-spoke architecture was implemented: local clients trained models, the server aggregated updates, and redistributed a refined global model for the next round. The authors experimented with varying local epoch counts and aggregation cadences, reflecting practical trade-offs between communication cost and convergence stability.

##### 5.5.1.6 Training configuration

Models were trained iteratively until convergence. Experiments compared centralized baselines, local-only training, and federated training under different client splits. Communication frequency and number of local epochs were systematically varied to evaluate their impact on accuracy and efficiency.

##### 5.5.1.7 Evaluation

Performance was measured primarily using area under the receiver operating characteristic curve (AUROC). Results demonstrated that global FL models consistently outperformed local-only models and closely matched centralized baselines. FedAvg remained stable across client heterogeneity, though imbalanced partitions slightly increased divergence risk when multiple local epochs preceded aggregation.

##### 5.5.1.8 Cost and sustainability

The framework reduced communication requirements relative to round-by-round synchronization by allowing multiple local epochs, though this introduced minor trade-offs in stability. No explicit energy or resource utilization metrics were reported, but the design reflected realistic hospital constraints such as variable network bandwidth and compute availability.

##### 5.5.1.9 Results and residual risk statement

The study confirmed that FL can achieve near-centralized performance on ICU mortality prediction without transferring raw patient records. However, residual risks remain: gradient updates may still leak information under strong adversaries, adversarial clients could poison updates, and the simulation on a single de-identified dataset does not fully reflect operational heterogeneity across live institutions.

##### 5.5.1.10 Lessons learned and recommendations

Vieira et al. (2025) demonstrate that FL provides a viable path for multi-institutional ICU prediction tasks, preserving utility while maintaining data locality. Yet, scaling to real hospital networks will require integration of additional PETs (e.g., differential privacy, secure aggregation, homomorphic encryption) to harden defenses. Future work should extend beyond simulated partitions to genuine multi-site deployments, incorporating governance agreements and standardized monitoring to ensure robustness and compliance.

#### 5.5.2 Cho et al.: Secure Federated GWAS with SMPC and HE at Biobank Scale

##### 5.5.2.1 Clinical context and objective

Genome-wide association studies (GWAS) are central to linking genetic variation with disease, but they require massive sample sizes for statistical power. Biobanks such as UK Biobank and eMERGE provide millions of genomes, yet strict governance rules make pooling raw data infeasible. Previous PET approaches had critical limitations: SMPC-only GWAS proved too slow at biobank scale, while HE-only frameworks could not support key workflows such as principal component analysis (PCA) or linear mixed models (LMMs). Cho et al. (86) introduced Secure Federated GWAS (SF-GWAS), a hybrid protocol combining SMPC and HE within a federated design, aiming to preserve the accuracy of pooled GWAS while scaling securely across hundreds of thousands of participants.

##### 5.5.2.2 Data and governance

SF-GWAS was evaluated on multiple disease cohorts, including lung cancer (*n* = 9,178), bladder cancer (*n* = 13,060), and age-related macular degeneration (AMD; *n* = 22,683). The framework was then scaled to eMERGE (*n* = 31,293) and UK Biobank (*n* = 275,812 for PCA; *n* = 409,547 for LMMs), and finally to a cross-biobank AMD study combining eMERGE, UK Biobank, and the International AMD Genomics Consortium (*n* = 111,807; >22 million variants). All analyses were performed without sharing raw genomic data. Harmonization of phenotype definitions and variant metadata was required across sites, underscoring governance challenges that accompany cryptographic deployments.

##### 5.5.2.3 Threat model

The protocol assumed an honest-but-curious adversary model. Data custodians never exposed raw records, and computations were protected using cryptographic primitives. However, defenses against fully malicious actors (e.g., misreporting or injecting corrupted updates) were not fully developed, leaving open avenues for adversarial exploitation in practical deployments.

##### 5.5.2.4 PET stack and rationale

SF-GWAS combines a federated architecture with a hybrid cryptographic workflow:

- Homomorphic encryption (HE) was used for large-scale linear algebra operations such as multiplying genotype matrices with phenotype vectors.
- Secure multiparty computation (SMPC) was employed for precision-critical operations such as division, normalization, and sign functions.

This division of labor balanced the computational strengths of HE with the exactness of SMPC, enabling full GWAS workflows (including QC, PCA, LMMs, and regression analyses) while avoiding the inefficiencies of single-method pipelines.

##### 5.5.2.5 System architecture

Each institution retained local genomic data and engaged in federated protocols orchestrated via a hub-and-spoke design. Local computations were performed under HE, while SMPC was invoked for cross-site nonlinear operations. The system was further supported by *sfkit*, a web-based toolkit for automating deployment across cloud or institutional servers, designed to reduce setup complexity.

##### 5.5.2.6 Training/inference configuration

The framework implemented end-to-end GWAS functionality: quality control pipelines, PCA for population structure, linear and logistic regression, and LMMs. Outputs were compared directly with plaintext pooled GWAS to benchmark fidelity.

##### 5.5.2.7 Evaluation

On moderate cohorts, SF-GWAS reduced runtime by more than an order of magnitude compared to Secure GWAS (S-GWAS), e.g., AMD analysis completed in 4.6 h vs. 64.3 h. Communication costs also fell three- to fourfold. At scale, the framework completed eMERGE analyses in <18 h and UK Biobank workflows in ∼5 days, feasible runtimes for collaborative genomics. Outputs matched centralized pooled analyses, identifying substantially more variants than site-specific or meta-analytic alternatives. In eMERGE, SF-GWAS replicated 71 of 73 Pan-UK Biobank associations, whereas local analyses identified only a handful.

##### 5.5.2.8 Cost and sustainability

While runtimes were significantly reduced, very large cohorts still required multi-day computation. Deployment also demanded careful metadata harmonization and substantial infrastructure, highlighting resource and governance hurdles for routine adoption.

##### 5.5.2.9 Results and residual risk statement

SF-GWAS demonstrated that hybrid cryptographic PETs can scale GWAS to biobank-sized datasets with near-centralized accuracy while keeping data siloed. Yet, residual risks persist: multi-day runtimes limit rapid analyses, metadata harmonization imposes administrative burden, and honest-but-curious assumptions leave gaps against malicious adversaries.

##### 5.5.2.10 Lessons learned and recommendations

Cho et al. (86) show that combining SMPC and HE within a federated design enables secure, scalable GWAS pipelines that preserve fidelity to pooled analyses. The work establishes feasibility for multi-site biobank studies but also highlights the need for: (i) runtime optimization for larger or latency-sensitive deployments; (ii) expanded PET stacks (e.g., DP or TEE layers) to harden against stronger adversaries; and (iii) standardized governance frameworks to streamline variant and phenotype harmonization. Extending these methods beyond GWAS to broader biobank analytics could accelerate secure, large-scale genomic discovery.

## 6 Residual risks and limitations

Even as privacy-enhancing technologies (PETs) mature, their deployment in biomedical AI remains limited by technical, operational, and institutional constraints. No single PET or hybrid configuration can completely eliminate privacy leakage or performance loss. Residual vulnerabilities persist throughout the model lifecycle, from training to deployment and inference, and are amplified in biomedical contexts where data are heterogeneous, sensitive, and often irreplaceable. Recognizing these limits is essential for setting realistic expectations and for designing complementary safeguards and governance strategies. The following subsections outline three main dimensions of residual risk: technical vulnerabilities, privacy–utility trade-offs, and the risks of misuse or overreliance.

### 6.1 Technical gaps and persistent vulnerabilities

Despite major advances, PETs for biomedical AI remain imperfect. Vulnerabilities arise from both design limitations and the ability of adversaries to adapt faster than defenses. Understanding these gaps is critical for developing robust and context-appropriate protections.

Federated learning (FL) maintains data locality but remains vulnerable to gradient leakage attacks that reconstruct sensitive patient data or images from shared updates, especially with small batch sizes or limited aggregation (35, 36). Secure aggregation reduces but cannot fully eliminate this risk, particularly when several malicious clients collude (4). Differential privacy (DP), although mathematically rigorous, is fragile under poor parameterization. When *ε* is too high, privacy guarantees degrade significantly in realistic biomedical settings (45). DP also tends to harm rare diseases and minority cohorts, where small sample sizes amplify the effect of noise (27).

Cryptographic PETs face other constraints. Secure multiparty computation (SMPC) provides strong protection but requires heavy computation and communication, limiting scalability for imaging or genomic analysis (49). Homomorphic encryption (HE) enables encrypted computation but remains far slower than plaintext operations, restricting its use mainly to inference (31). These inefficiencies limit feasibility for near real-time analysis or large-scale studies.

Post-deployment vulnerabilities persist. Most PET research focuses on training, yet inference-time exposure remains underexplored. Deployed models can still leak patient information through membership inference or model inversion, especially when trained on small or sensitive cohorts (6, 7). These risks emphasize the importance of defenses that protect models across their entire lifecycle.

Hybrid PETs attempt to address individual weaknesses but introduce composability risks. For example, adding DP noise to HE-protected outputs without proper calibration can distort data and weaken formal guarantees (60). Without rigorous frameworks to analyze how PETs interact, hybrid pipelines may unintentionally undermine their own protections.

In summary, existing PETs leave biomedical AI models exposed to risks during training, deployment, and inference. Reducing these vulnerabilities will require adaptive defenses, scalable cryptographic protocols, and new frameworks that validate hybrid pipelines. Until such progress is achieved, PET deployment in biomedical AI will remain limited by issues of robustness, efficiency, and trust.

### 6.2 Privacy–utility trade-offs

The use of PETs in biomedical AI always involves balancing privacy guarantees against model performance. Stronger safeguards reduce disclosure risks but often lower accuracy, generalizability, and fairness. This tension is especially pronounced in biomedical domains, where datasets are small, imbalanced, or dominated by rare conditions.

Differential privacy (DP) illustrates this tension most clearly. Adding noise to gradients or outputs can bound information leakage but also reduce predictive accuracy (23). The effect is strongest in low-signal domains such as early cancer detection or rare variant classification (30). The performance loss is not uniform, as underrepresented groups typically experience steeper degradation, which can worsen health disparities (27).

Federated learning (FL) allows data to remain local but introduces new utility challenges. Differences in dataset size, feature distribution, and hardware can destabilize training and bias the global model (28). When FL is combined with other PETs such as secure aggregation or DP, these instabilities may increase, offsetting much of the accuracy gained from federation.

Cryptographic PETs such as HE and SMPC maintain fidelity in theory but incur severe computational costs in practice. To remain tractable, developers must reduce model depth, training iterations, or feature dimensions (31, 49). These compromises limit performance in complex tasks such as imaging and multi-omics analysis.

Hybrid configurations try to balance these trade-offs by combining PETs with complementary strengths. HE can secure data aggregation, while DP noise at the output layer reduces disclosure risk. Although this layering strengthens privacy, the cumulative overhead often harms utility, especially when parameters are tuned too conservatively (60). Poor calibration can lead to models that are private but not clinically useful.

The privacy–utility trade-off cannot be eliminated but can be managed. The optimal balance depends on the intended use, the regulatory environment, and institutional risk tolerance. Achieving it requires empirical evaluation of PET setups under realistic biomedical conditions and standardized benchmarks that jointly measure privacy leakage and predictive accuracy. Without evidence-based calibration, PETs risk either exposing sensitive data or degrading their clinical value.

### 6.3 Misuse and overreliance on PETs

While PETs are essential for protecting biomedical data, misuse or overreliance can undermine their effectiveness. Excess confidence in technical safeguards often leads to a false sense of security and leaves systemic vulnerabilities unresolved.

A common misuse is assuming that a single PET is sufficient. Using FL without additional safeguards such as secure aggregation or DP exposes models to gradient inversion attacks (35, 36). Similarly, applying DP without proper tuning can either cripple model performance or provide little real protection if *ε* is set too high (45). In both cases, compliance may appear to be met while privacy protection remains weak.

PETs are also used beyond their validated scope. HE is well suited for small-scale inference but remains impractical for deep learning at scale due to high computational cost (31). SMPC performs well for smaller analyses but scales poorly for high-dimensional genomic or imaging data, where performance quickly declines (49). Misuse in these contexts wastes resources and can interrupt clinical workflows.

A deeper issue arises when PETs are deployed in isolation rather than as part of broader security and governance structures. Cryptographic or statistical safeguards cannot prevent breaches from compromised endpoints, insider misuse, or poor access control (30). A model trained with DP may still leak sensitive data if downstream systems allow unrestricted queries, and federated networks can be corrupted by malicious clients (27).

Institutions sometimes treat PETs mainly as compliance tools. While PET adoption supports alignment with GDPR and HIPAA, regulators emphasize that technical safeguards must be part of comprehensive privacy programs that also include organizational and procedural controls (87). Treating PETs as complete compliance guarantees increases the risk of both privacy breaches and legal exposure.

Effective protection requires integrating PETs within a broader framework that includes clear threat models, validated configurations, and complementary safeguards such as access control, audit logging, and model monitoring. Only through such coordinated strategies can PETs provide reliable and sustainable privacy protection in biomedical AI.

## 7 Conclusion and future directions

This section synthesizes findings in direct response to the four guiding research questions (RQ1–RQ4), linking lifecycle threats, PET mapping, infrastructural feasibility, and residual risks. Privacy-enhancing technologies (PETs) have emerged as essential tools for reconciling the tension between data utility and confidentiality in biomedical AI. Differential privacy, secure multiparty computation, homomorphic encryption, and federated learning each offer distinctive strengths, yet none can independently satisfy the diverse requirements of clinical and research environments. Biomedical datasets are complex, heterogeneous, and sensitive, demanding solutions that protect against sophisticated adversaries while preserving the accuracy and interpretability required for high-stakes decision-making. In relation to RQ1, this review shows that privacy threats evolve dynamically across the model lifecycle, shifting from training-phase gradient leakage to inference-time membership and inversion risks, which underscores why protections must be lifecycle-aware. This review has highlighted both the progress made and the persistent limitations of PETs, underscoring that privacy protection remains an evolving challenge rather than a solved problem.

Hybrid and emerging PET combinations represent a promising path forward. By layering cryptographic methods with statistical guarantees and federated designs, these approaches reduce reliance on any single defense and improve resilience across different stages of the model lifecycle. Case studies demonstrate that federated learning can scale effectively to critical care prediction, while hybrid cryptographic frameworks can enable secure genome-wide association studies at biobank scale. At the same time, composability remains an unresolved issue. Sequentially combining PETs without rigorous calibration can weaken rather than strengthen guarantees, and excessive layering may impair clinical usability. Addressing these tensions will require not only technical innovation but also systematic benchmarking under biomedical workloads that capture privacy, utility, fairness, and performance in an integrated way. These findings provide an answer to RQ2 by clarifying that while PETs cover distinct aspects of biomedical privacy, their true potential lies in carefully engineered hybrid deployments.

Looking ahead, the field must prioritize the development of modular and composable PET stacks that can adapt flexibly to varying regulatory, infrastructural, and clinical contexts. Advances in hardware acceleration, confidential computing environments, and verifiable computation hold particular promise for reducing the overhead of cryptographic approaches. Meanwhile, privacy-preserving machine learning must expand to modalities that are increasingly central to biomedical AI, including multimodal integration, large language models trained on clinical text, and high-throughput genomic pipelines. Equally important will be the creation of standardized protocols for privacy budget management and the establishment of benchmarks that enable fair comparison across methods and domains. These recommendations address RQ3 by emphasizing the infrastructural and deployment considerations required for PET integration into real-world biomedical workflows.

The future of biomedical AI will depend on embedding privacy as a foundational design principle rather than a retrofitted safeguard. PETs provide the mathematical and technical foundations for this shift, but their success will ultimately hinge on integration with governance structures, institutional practices, and clinical workflows. A sustainable path forward lies in building PET-enabled ecosystems that are secure by design, clinically validated, and responsive to evolving societal expectations. Achieving this vision will require collaboration across technical, clinical, and regulatory communities, ensuring that biomedical AI advances not only in power and scope but also in its respect for the privacy and trust of patients it is designed to serve. In relation to RQ4, these conclusions highlight that residual risks such as fairness concerns, inference-time leakage, and overreliance on PETs as compliance proxies persist, and that the next decade of research must tackle these challenges in tandem with governance reforms.

## Data Availability

The original contributions presented in the study are included in the article; further inquiries can be directed to the corresponding authors.

## Appendix A. Database-Specific Search Strings

**Table:**

DATABASE	SEARCH STRING
PUBMED	((“differential privacy"[Title/Abstract] OR “federated learning"[Title/Abstract] OR “secure multiparty computation"[Title/Abstract] OR “secure multi-party computation"[Title/Abstract] OR “homomorphic encryption"[Title/Abstract] OR “synthetic data"[Title/Abstract] OR “synthetic data generation"[Title/Abstract] OR “trusted execution environment"[Title/Abstract] OR “zero-knowledge proof"[Title/Abstract]) AND (biomedical[Title/Abstract] OR clinical[Title/Abstract] OR healthcare[Title/Abstract] OR “electronic health record"[Title/Abstract] OR EHR[Title/Abstract] OR “medical imaging"[Title/Abstract] OR genomic*[Title/Abstract] OR biobank*[Title/Abstract] OR wearable*[Title/Abstract] OR “clinical decision support"[Title/Abstract]) AND (privacy[Title/Abstract] OR “privacy leakage"[Title/Abstract] OR “re-identification"[Title/Abstract] OR “membership inference"[Title/Abstract] OR “model inversion"[Title/Abstract] OR “attribute inference"[Title/Abstract] OR deployment[Title/Abstract] OR implementation[Title/Abstract])) AND (“2015/01/01"[Date - Publication]: “2025/08/31"[Date - Publication])
IEEE XPLORE	(“All Metadata":"differential privacy” OR “All Metadata":"federated learning” OR “All Metadata":"secure multiparty computation” OR “All Metadata":"secure multi-party computation” OR “All Metadata":"homomorphic encryption” OR “All Metadata":"synthetic data” OR “All Metadata":"trusted execution environment” OR “All Metadata":"zero-knowledge proof”) AND (“All Metadata":biomedical OR “All Metadata":clinical OR “All Metadata":healthcare OR “All Metadata":"electronic health record” OR “All Metadata":EHR OR “All Metadata":"medical imaging” OR “All Metadata":genomics OR “All Metadata":biobank OR “All Metadata":wearable) AND (“All Metadata":privacy OR “All Metadata":"re-identification” OR “All Metadata":"membership inference” OR “All Metadata":"model inversion” OR “All Metadata":"attribute inference” OR “All Metadata":deployment OR “All Metadata":"clinical implementation”), filtered to 2015–2025.
ACM DIGITAL LIBRARY	[[All: “differential privacy”] OR [All: “federated learning”] OR [All: “secure multiparty computation”] OR [All: “secure multi-party computation”] OR [All: “homomorphic encryption”] OR [All: “synthetic data”] OR [All: “trusted execution environment”] OR [All: “zero-knowledge proof”]] AND [[All: biomedical] OR [All: clinical] OR [All: healthcare] OR [All: “electronic health record”] OR [All: EHR] OR [All: “medical imaging”] OR [All: genomics] OR [All: biobank] OR [All: wearable]] AND [[All: privacy] OR [All: “re-identification”] OR [All: “membership inference”] OR [All: “model inversion”] OR [All: “attribute inference”] OR [All: deployment] OR [All: “clinical implementation”]], filtered to 2015–2025.
SCOPUS	TITLE-ABS-KEY((“differential privacy” OR “federated learning” OR “secure multiparty computation” OR “secure multi-party computation” OR “homomorphic encryption” OR “synthetic data” OR “synthetic data generation” OR “trusted execution environment” OR “zero-knowledge proof”) AND (biomedical OR clinical OR healthcare OR “electronic health record” OR EHR OR “medical imaging” OR genomic* OR biobank* OR wearable* OR “clinical decision support”) AND (privacy OR “privacy leakage” OR “re-identification” OR “membership inference” OR “model inversion” OR “attribute inference” OR deployment OR “clinical implementation”)) AND PUBYEAR > 2014 AND PUBYEAR < 2026
